# Maternally transferred thyroid hormones and life‐history variation in birds

**DOI:** 10.1111/1365-2656.13708

**Published:** 2022-05-07

**Authors:** Bin‐Yan Hsu, Veli‐Matti Pakanen, Winnie Boner, Blandine Doligez, Tapio Eeva, Ton G. G. Groothuis, Erkki Korpimäki, Toni Laaksonen, Asmoro Lelono, Pat Monaghan, Tom Sarraude, Robert L. Thomson, Jere Tolvanen, Barbara Tschirren, Rodrigo A. Vásquez, Suvi Ruuskanen

**Affiliations:** ^1^ Department of Biology University of Turku Turku Finland; ^2^ Ecology and Genetics Research Unit University of Oulu Oulu Finland; ^3^ Department of Biological and Environmental Sciences University of Gothenburg Gothenburg Sweden; ^4^ Institute of Biodiversity, Animal Healthy and Comparative Medicine University of Glasgow Glasgow UK; ^5^ Department of Biometry and Evolutionary Biology, CNRS UMR 5558 Université de Lyon 1 Villeurbanne France; ^6^ Groningen Institute for Evolutionary Life Sciences (GELIFES) University of Groningen Groningen The Netherlands; ^7^ Biology Department, Natural Sciences and Mathematics Faculty Jember University of Indonesia Jember Indonesia; ^8^ Fitzpatrick Institute of African Ornithology, DST‐NRF Centre of Excellence University of Cape Town Cape Town South Africa; ^9^ Centre for Ecology and Conservation University of Exeter Exeter UK; ^10^ Instituto de Ecología y Biodiversidad, Departamento de Ciencias Ecológicas, Facultad de Ciencias Universidad de Chile Santiago Chile; ^11^ Department of Biological and Environmental Sciences University of Jyväskylä Jyväskylä Finland

**Keywords:** Aves, developmental mode, life‐history variation, maternal hormone transfer, migration, pace of life, phylogenetic comparative analysis, yolk hormones

## Abstract

1. In vertebrates, thyroid hormones (THs) play an important role in the regulation of growth, development, metabolism, photoperiodic responses and migration. Maternally transferred THs are important for normal early phase embryonic development when embryos are not able to produce endogenous THs. Previous studies have shown that variation in maternal THs within the physiological range can influence offspring phenotype.
2. Given the essential functions of maternal THs in development and metabolism, THs may be a mediator of life‐history variation across species.
3. We tested the hypothesis that differences in life histories are associated with differences in maternal TH transfer across species. Using birds as a model, we specifically tested whether maternally transferred yolk THs covary with migratory status, developmental mode and traits related to pace‐of‐life (e.g. basal metabolic rate, maximum life span).
4. We collected un‐incubated eggs (*n* = 1–21 eggs per species, median = 7) from 34 wild and captive bird species across 17 families and six orders to measure yolk THs [both triiodothyronine (T3) and thyroxine (T4)], compiled life‐history trait data from the literature and used Bayesian phylogenetic mixed models to test our hypotheses.
5. Our models indicated that both concentrations and total amounts of the two main forms of THs (T3 and T4) were higher in the eggs of migratory species compared to resident species, and total amounts were higher in the eggs of precocial species, which have longer prenatal developmental periods, than in those of altricial species. However, maternal yolk THs did not show clear associations with pace‐of‐life‐related traits, such as fecundity, basal metabolic rate or maximum life span.
6. We quantified interspecific variation in maternal yolk THs in birds, and our findings suggest higher maternal TH transfer is associated with the precocial mode of development and migratory status. Whether maternal THs represent a part of the mechanism underlying the evolution of precocial development and migration or a consequence of such life histories is currently unclear. We therefore encourage further studies to explore the physiological mechanisms and evolutionary processes underlying these patterns.

## Graphical abstract

Exploring the interspecific variation in maternal yolk thyroid hormones across more than 30 species of birds, the authors' results suggest that the interspecific variation of maternal thyroid hormones in birds is related to their migratory status and developmental mode.

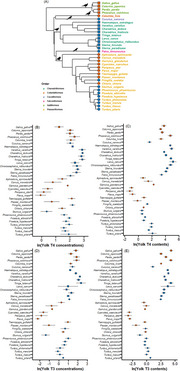

## 1 INTRODUCTION

Thyroid hormones (THs) regulate many aspects of growth and development as well as metabolism in both young and adults (Darras, 2019; McNabb & Darras, 2015; Moog et al., 2017; Mullur et al., 2014). Across vertebrates, the thyroid gland predominantly produces thyroxine (T4), which can be converted into triiodothyronine (T3). Because T3 has a much higher affinity with receptors than T4, and therefore exerts most of the receptor‐mediated effects, T3 is usually considered the ‘biologically active form’ of THs, whereas T4 is considered a prohormone (Darras et al., 2015). Although some independent functions of T4 have been proposed (e.g. via non‐genomic actions, Davis et al., 2016), the conversion of T4 to T3 by deiodinase enzymes in the brain and peripheral tissues is a vital mechanism that regulates the intracellular availability of T3 and hence the local TH function (Darras et al., 2015; Darras & van Herck, 2012). For simplicity, in this article, we use ‘THs’ when talking about the general functions of thyroid hormones, and T3 or T4 when talking about one specific form.

The THs of maternal origin are critical in the regulation of development during the very early embryonic stage. In placental mammals, including humans, maternal THs can access the developing embryos across the placenta (Morreale de Escobar et al., 2004; Patel et al., 2011). Medical studies have recognized the indispensability of maternally transferred THs (hereafter maternal THs) for normal, healthy foetal development during early pregnancy (Moog et al., 2017; Morreale de Escobar et al., 2004). In oviparous animals, such as birds, maternal THs are transferred and deposited into egg yolks during egg formation, from where they can reach the embryos and influence developmental process, and eventually offspring phenotype (Ruuskanen & Hsu, 2018). Although early embryos are not yet able to produce endogenous THs, they already express necessary molecules, including TH receptors and deiodinases, to respond to maternal THs in less than 4 days of incubation (Flamant & Samarut, 1998; van Herck et al., 2012, 2015; Too et al., 2017; Ruuskanen, Hukkanen, et al., 2021). Experimental manipulation of THs during early embryo development (albeit often using supraphysiological doses) has demonstrated influences on development and gene expression in chickens *Gallus gallus domesticus* (Flamant & Samarut, 1998; Darras et al., 2009; Darras, 2019), Japanese quail *Coturnix japonica* (Wilson & McNabb, 1997), frogs *Xenopus tropicalis* (Duarte‐Guterman et al., 2010) and various fishes (reviewed in Brown et al., 2014). For example, yolk TH injection reduced the expression of the transporter OATP1C1 mRNA but increased the expression of another transporter MCT8 in 4‐day‐old embryonic chicken brain (van Herck et al., 2012). With the molecular machinery ready to respond to maternally transferred THs during a time window when endogenously produced THs are not yet available, maternal THs may therefore have organizational effects on offspring and represent different facets of significance from the endogenous THs in later life.

Variation in maternal TH transfer within the physiological range (i.e. no clinical or subclinical TH disorder that would induce maldevelopment) can also influence offspring phenotype. In humans, correlative studies have found that variation in maternal THs positively correlates with infant birth weight (Medici et al., 2013) and the IQ of children (Korevaar et al., 2016). In birds, a handful of studies also provide evidence that experimentally elevated yolk THs that mimic higher maternal transfer influence offspring growth and physiology, and therefore may have fitness consequences, although mixed effects have been reported (Hsu et al., 2017; Hsu, Doligez, et al., 2019; Ruuskanen, Darras, Visser, & Groothuis, 2016; Sarraude et al., 2020a). Together, these studies suggest that variation in maternal TH transfer may have ecological and evolutionary consequences in vertebrates. Nevertheless, maternal THs in wild animals have received little attention (Ruuskanen & Hsu, 2018), particularly in mammals and reptiles, and all previous studies (see above) were conducted at the within‐species level. Here, using birds as a model, we aim to explore, for the first time, interspecific variation in maternal TH transfer across species and its associations with life‐history variation by a phylogenetic comparative approach.

Given the diverse and far‐reaching effects of THs on metabolic rate regulation (Mullur et al., 2014), thermogenesis and thermoregulation (Mullur et al., 2014; Price & Dzialowski, 2018; Ruuskanen, Hsu, & Nord, 2021), tissue differentiation, growth and maturation (McNabb & Darras, 2015; Wilson & McNabb, 1997), species with different life histories may show associated differences in their TH physiology. For example, Jetz et al. (2008) found that migratory birds generally have higher basal metabolic rates (BMRs) than resident species. Although the high BMRs in migratory birds may be driven by their generally colder breeding areas in the high latitudes, high BMR itself may be associated with higher levels of circulating THs (e.g. Chastel et al., 2003; Elliott et al., 2013; Welcker et al., 2013) and may result in higher transfer of maternal THs to egg yolks. Previous studies have also suggested the roles of THs on migration in birds (e.g. Pant & Chandola‐Saklani, 1993; Pérez et al., 2016) and fish (e.g. Kitano et al., 2010). Moreover, THs interact with other endocrine axes. Specifically, accumulating evidence suggests that THs are involved in gonadal differentiation and maturation across vertebrates (birds: Flood et al., 2013; Sechman, 2013; amphibians: Duarte‐Guterman et al., 2014; and fish: Rodrigues et al., 2021). It is well‐established that in both mammals and birds, THs play a central role in regulating seasonal breeding (Dardente et al., 2014; Nishiwaki‐Ohkawa & Yoshimura, 2016; Shinomiya et al., 2014). THs and glucocorticoids (stress hormones) also have synergistic effects during major life stage transition across vertebrates, such as the smoultification in salmonid fish, metamorphosis in amphibians and fish, hatching in birds and birth in mammals (reviewed in Wada, 2008; Watanabe et al., 2016; Rousseau et al., 2021). Therefore, THs could also play a mediating role in reproductive investment and shape the trade‐offs between survival and reproduction and between current and future reproduction, as has been found for glucocorticoids (Bókony et al., 2009; Casagrande et al., 2018; Hau et al., 2010; Vitousek et al., 2019).

All in all, known TH functions imply a potential role in mediating life‐history variation. Interspecific differences in life history‐associated TH physiology may have downstream effects on maternal TH transfer, reflecting the differences in blood circulating TH levels. Alternatively, because of the essential function of maternal THs in early embryonic development, differences in yolk THs could also arise as a result of adaptive evolution. We therefore generated three specific and testable hypotheses about how life‐history variation may be associated with interspecific variation in maternally transferred THs, using birds as a model system:

First, studies in wild birds have reported positive correlations between circulating T3 and BMR or RMR (resting metabolic rate; Chastel et al., 2003; Elliott et al., 2013; Welcker et al., 2013). Since migratory birds have higher BMRs than resident birds (Jetz et al., 2008), they may have been selected for higher circulating THs and consequently higher maternal TH transfer. Moreover, THs have been suggested to regulate migration (e.g. Pant & Chandola‐Saklani, 1993; Pérez et al., 2016). We therefore hypothesize that migratory species deposit higher TH levels (higher concentrations and/or larger total amounts) in egg yolks than resident species (Hypothesis 1).

Second, birds exhibit a continuous spectrum from altricial to precocial development (Starck & Ricklefs, 1998). Across the spectrum, altricial and precocial birds show distinct ontogenetic trajectories (e.g. McNichols & McNabb, 1988, reviewed inde Groef et al., 2013; McNabb, 2006): At hatching, precocial birds have a more advanced development in visual and locomotory abilities and thermoregulation compared to altricial species (Price & Dzialowski, 2018; Starck & Ricklefs, 1998). Also, in precocial birds, thyroid function already starts in the middle of incubation and the TH levels in thyroid gland and blood circulation both peak around hatching (de Groef et al., 2013; McNabb, 2006). By contrast, thyroid function of altricial species starts days after hatching, and their circulating blood THs gradually increase towards the adult level (McNabb, 2006; de Groef et al., 2013). The lengths of developmental periods are also longer in precocial compared to altricial species (Starck & Ricklefs, 1998, see also Supplementary materials). Based on the distinct differences in ontogeny and assuming maternal THs are related to the more advanced prenatal development, we hypothesize that precocial species deposit higher TH levels (higher concentrations and/or larger total amounts) in egg yolks than altricial species, even after controlling for body size (Hypothesis 2). Alternatively, because altricial species are not able to produce endogenous THs until after hatching, one might instead postulate that altricial species would deposit higher TH levels in the egg yolks to support embryonic development. The lengths of developmental periods are also expected to correspondingly correlate with the level of maternal yolk THs.

Third, because of resource trade‐off and allocation, life‐history variation usually exhibits a fast–slow pace‐of‐life continuum, which may be mediated by metabolic rates (Brown et al., 2004; Dammhahn et al., 2018; Healy et al., 2019). Based on THs' positive correlation with metabolic rates, we hypothesize that the species living a faster pace‐of‐life deposit higher concentrations and/or larger amounts of yolk THs (Hypothesis 3). Specifically, we predicted a positive correlation between yolk THs and a species' fecundity (clutch size and number of clutches per year), BMR and growth rate, but a negative correlation with a species' maximum life span and age at sexual maturity.

To test these three hypotheses, we collected and measured THs in eggs before incubation (to ensure no TH metabolism by embryos) from 34 wild and captive bird species varying in body masses, migratory status, developmental modes and pace‐of‐life‐related traits and used a Bayesian phylogenetic mixed‐effects models to test our hypotheses.

## 2 MATERIALS AND METHODS

### 2.1 Sample collection

We collected un‐incubated eggs from 34 species of birds across 17 families and six orders (*n* = 1–21 eggs per species, median = 7, Table S1). Eggs from wild species (*n* = 28) were collected by extensive nest searches in the known breeding habitats or from nest‐box populations, while eggs from six species came from captive animals. We aimed at collecting eggs before clutch completion (i.e. when found, the eggs were cold and clutch was not complete). In order to avoid pseudo‐replication (i.e. within‐clutch non‐independence in yolk THs) and ensure minimal impact on individual reproductive success, we only collected one, randomly sampled egg per clutch for each species. All eggs were frozen and stored at −20°C on the day of collection until further analysis. Eggs of wild species were collected under licences from the environmental authorities in each country: Finland, VARELY/665/2016, POPELY/61/2016, VARELY/63/2016, VARELY/412/2016, VARELY/6085/2016 and Finnish Wildlife Agency 84/2016; Chile, Agricultural Ministry 404/2017; and Sweden, National Board for Laboratory Animals. Captive species were housed and eggs were collected following all national and international guidelines respectively. Eggs of homing pigeons *Columba livia domesticus*, domestic chicken *Gallus gallus domesticus*, grey partridges *Perdix perdix* and pheasants *Phasianus colchicus* were acquired from local breeders in Finland and no licence was needed. The eggs of Japanese quail *Coturnix japonica*, rock pigeons *C. l. livia* and red junglefowl *G. g. bankiva* were collected from the maintained colonies at the University of Turku, Finland (ESAVI/1018/04.10.07/2016 for quail) and the University of Groningen, the Netherlands (DEC No. 5635E, 5635G for rock pigeons; and 6710B‐001 for red junglefowl). The zebra finch *Taeniopygia guttata* eggs were unfertilized eggs that were not under any experimental procedure at the University of Glasgow, UK, and therefore no licence was required. Most of the eggs (both wild and captive species) were collected during 2016–2017, except those of the collared flycatcher (*Ficedula albicollis*, collected in 2011), rock pigeons (collected in 2014) and red junglefowl (collected in 2015).

### 2.2 Yolk TH analysis

We extracted THs from the egg yolks following a previously described protocol (Ruuskanen, Darras, de Vries, et al., 2016). In brief, we weighed egg yolks and homogenized them in MQ water and a subsample of the yolk‐MQ mixture (*c*. 300 mg) was used for TH extraction by methanol and chloroform. Before extraction, samples were spiked with a known amount of ^13^C_12_‐T4 to track recovery. Samples then went through chloride‐form anion exchange resin (Bio‐Rad) for purification. The final products were re‐dissolved in 0.01% NH_3_ buffer and yolk T3 and T4 were simultaneously measured using a validated nano‐flow liquid chromatography‐mass spectrometry (LC–MS) protocol (Ruuskanen et al., 2018). Our protocols extracted and measured the total T3 and T4 (i.e. both free, unbound hormones and bound hormones). The LC–MS/MS method has previously been validated for several Galliform and Passeriform species (Ruuskanen et al., 2018). Because this method does not rely on antibody specificity, and we saw no signs of any potential problems during the measurements (e.g. wrong retention time), we are confident that the method works for all our species. Across all samples, the average recovery varied from 30.90% to 52.01%, and there was no pattern of systematic bias across species (Figure S1). The measurements were normalized against an internal standard, calibrated by a standard line (*R* ^2^ ≥ 0.99) and corrected for recovery. More details for TH extraction and the LC–MS methods are provided in Supplementary methods. All TH concentrations were expressed as pg/mg yolk. The total amounts of THs per yolk were calculated by multiplying the concentration with the total yolk mass and expressed as ng/yolk. Although yolk THs might degrade over time under storage, our assessment suggested that the storage effects appeared to have limited influence in our samples and unlikely to bias our results (see Supplementary Methods)

### 2.3 Life‐history traits

We collected life‐history trait values for the species from which we measured yolk TH levels from the literature. All literature sources, along with the trait values used in our analyses, are presented in the Supplementary Data.

Species' body mass was collected from the amniote life‐history database compiled by Myhrvold et al. (2015). To test whether a species' developmental mode explains yolk TH variation, we categorized our species as either precocial or altricial species based on Cramp (1977‐1994) and Starck and Ricklefs (1998). The four semi‐precocial species, which all belong to the family Laridae (gulls and terns), were pooled with precocial species and the one semi‐altricial species (the kestrel, *Falco tinnunculus*) was pooled with altricial species. In addition to further explore whether the variation in yolk THs may be at least partly attributed to differences in developmental duration between precocial and altricial species, we collected data on incubation duration and the age at fledging (i.e. number of days from hatching to fledging) from Myhrvold et al. (2015) for each species to represent the length of the prenatal and postnatal developmental period respectively. When such data were not available in Myhrvold et al. (2015), we consulted other literature (documented in Supplementary Data).

To test whether a species' migratory status explains yolk TH variation, we categorized each species as either migratory or resident, based on Lehikoinen et al. (2003), McNab (2009) and Pap et al. (2015). When the categorization was not consistent in these references, we determined the migratory status based on the population from which we collected eggs.

To explore associations between yolk THs and pace‐of‐life‐related traits, we collected the following traits:

- Growth rate, measured as the logistic growth rate constants from Starck and Ricklefs (1998) for better comparability. More recent literature was also consulted. When data from multiple sources were available, we calculated mean value. All literature we consulted are documented in Supplementary Data.
- Clutch size and number of clutches per year were collected mostly from Myhrvold et al. (2015), supplemented by other literature documented in Supplementary Data to represent a species' fecundity.
- Basal metabolic rates (BMRs), mainly obtained from McKechnie et al. (2006) and McNab (2009), supplemented by other literature documented in Supplementary Data.
- Maximum life span and age at sexual maturity were obtained from the database AnAge (Tacutu et al., 2013). We chose maximum life span rather than other measures, such as median life span because of its much higher availability (de Magalhães et al., 2007; Healy et al., 2014). Maximum life span is also mostly determined by intrinsic factors and less sensitive to extrinsic cause of death, such as predation, and, therefore, is an arguably better measure for the true potential of longevity of a species (Barja, 2013; Vágási et al., 2019).

When consulting the literature, we assumed that the trait values are representative of the traits from wild populations. For all captive species (see above and the Supplementary Data), we used the trait values from the literature based on the specific colony from which the eggs were collected when available (e.g. the rock pigeons and zebra finches). In other cases, we assumed that the literature data are representative (e.g. for pheasants and grey partridges). Among our captive species, four are considered domesticated (domestic chicken, Japanese quail, homing pigeon and zebra finch), for which we additionally checked whether the trait values we obtained from the literature are relevant. These species may have highly different trait values compared to their wild ancestors. For example, the body mass of homing pigeons is known to be much larger than that of the wild‐type rock pigeons. When the literature data were questionable, we consulted known experts or left it as unavailable data (e.g. clutch sizes for domesticated chicken and Japanese quail are not determinable because they have been selected to lay eggs continuously).

In addition, whether a species is captive or wild was also taken into account because captive environments differ in many aspects from wild environments, such as in food availability, predation risk, spatial confinement and regular human disturbance (Beaulieu, 2016; Mason, 2010), all of which may influence TH physiology (e.g. Angelier et al., 2016). Because all domesticated species in our data set are captive, domestication (yes or no) was not included in statistical analyses but was nevertheless documented in the Supplementary Data.

### 2.4 Phylogenetic mixed models

#### 2.4.1 Brownian motion versus Ornstein‐Uhlenbeck process

When analysing interspecific data, the residuals are most likely non‐independent because of shared evolutionary history, which must be accounted for in the statistical model (Felsenstein, 1985). However, the true underlying evolutionary pattern is usually unknown. Because knowledge on yolk THs in wild birds is extremely limited, identifying a most likely evolutionary pattern of yolk THs is premature and beyond the scope of this paper. As such, we only assessed the two most commonly considered evolutionary models, Brownian motion (BM, Felsenstein, 1985) and a single‐optimum Ornstein–Uhlenbeck (OU) process (Hansen & Martins, 1996), to determine how the phylogenetic non‐independence should be dealt with in our analysis. The BM model assumes that trait differences accrue over time and therefore are proportional to the branch lengths between species (Felsenstein, 1985). The OU process builds on a BM model, with an additional parameter to describe a force of stabilizing selection (*α*) that pulls the trait value back towards an optimum (Hansen & Martins, 1996). Using the r package geiger (Pennell et al., 2014), the BM model was favoured, as the Pagel's *λ* for yolk T3 and T4 are both at bound (T3, *λ* = 1; T4, *λ* = 0.999), consistent with BM performance (Freckleton et al., 2002). The assessment also suggested the pulling force *α* is very small (T3, *α* = 0.006; T4, *α* = 0.012), rendering the model essentially equivalent to a BM process (Hansen & Martins, 1996). We therefore considered BM an appropriate assumption for our phylogenetic mixed models to control for phylogenetic non‐independence.

#### 2.4.2 Model specifications

We used phylogenetic mixed models (Housworth et al., 2004) to test our hypotheses and chose a Bayesian approach following Hadfield and Nakagawa (2010) with the r package mcmcglmm (Hadfield, 2010). Yolk T3 and T4 concentrations (pg/mg yolk) as well as total contents (ng/yolk) were ln‐transformed and standardized and used as dependent variables in separated univariate models. Because yolk T3 and T4 may exhibit different patterns of variation (as found e.g. in great tits, Hsu, Verhagen, et al., 2019), we analysed T3 and T4 with separate models. In all models, species body mass and captivity status (captive versus wild) were always included, along with the life‐history traits of interest, as fixed factors in the respective models (see below and Table 1). In order to facilitate model fitting, body mass was ln‐transformed and standardized and all two‐level categorical variables were dummy‐coded as −0.5 and 0.5 (Table S2). The life‐history traits that are known to be associated with body mass (BMR, growth rate, maximum life span, age at sexual maturity) were first corrected for body mass and phylogenetic relatedness among species using the r package phytools (Revell, 2009, 2012). BMR data were first converted to mass‐specific BMR (KJ hr^−1^ g^−1^) and ln‐transformed and corrected for body mass and phylogeny as pointed out previously.

**TABLE 1:** Model, phylogeny and datasets implemented in this study

Model set	Phylogeny and dataset	Fixed factors	Remark	Hypotheses tested
1	Full dataset with all 34 species	Developmental mode + migration + body mass + maximum life span + captivity		Hypotheses 1, 2, part of 3 (maximum life span)
2a	Excluding domesticated Japanese quail and domesticated chicken^a^	Developmental mode + migration + body mass + incubation duration + age at fledging + captivity	Age at fledging was missing for Japanese quail and domesticated chicken	Hypothesis 2
2b		Migration + body mass + incubation duration + age at fledging + captivity		
3	Excluding domesticated Japanese quail and domesticated chicken^a^	Developmental mode + migration + body mass + clutch size + clutches per year + captivity	Domesticated quail and chicken do not lay clear ‘clutches’ of eggs	Hypothesis 3 (fecundity traits)
4	Excluding *Gallus gallus*	Developmental mode + migration + body mass + age at sexual maturity + captivity	Age at sexual maturity data were missing for both jungle fowl and chicken	Hypothesis 3 (age at sexual maturity)
5	28 species with BMR data	Developmental mode + migration + body mass + BMR + captivity		Hypothesis 3 (BMR)
6	29 species with growth rate data	Developmental mode + migration + body mass + growth rate + captivity		Hypothesis 3 (growth rate)

a In Model sets 2 and 3, both Japanese quail and domesticated chicken were removed from the ‘dataset but only *Coturnix japonica* was removed from the phylogeny because *Gallus gallus* was still needed in the phylogeny for the data from red junglefowl.

Because data on life‐history traits were not available for all species, we defined several sets of models depending on data availability, listed in Table 1. Each model and dataset were thus pruned for the specific set of species where data were available. Unlike placental animals where mothers may continuously transfer maternal hormones to the embryo throughout the gestational period, the total amounts of maternal hormones in egg yolks are determined during yolk formation and mothers cannot supplement more thereafter. Therefore, the total contents of hormones represent an initial maximum hormone availability and, albeit unclear, probably represents different biological significance from the concentration. We therefore analysed both concentrations (pg/mg yolk) and total contents (ng/yolk) of T3 and T4 in separate model sets (four models per set in total).

Overall, the Model set 1 may be viewed as the general model set that tests our hypotheses using the traits for which data are available for all the 34 species. Model set 2 tests for covariation between yolk THs and developmental duration (i.e. incubation duration and age at fledging). Because precocial species have larger values for these two traits (Figure S4), we ran this set of models with and without developmental mode included (Model set 2a and 2b, respectively, Table 1). Model sets 3–6 specifically test for associations between THs and fecundity, age at sexual maturity, BMR and growth rate, using the respective phylogeny and data subsets.

In all models, the phylogenetic relatedness among all species (represented by the branch lengths) was treated as random effects, following Hadfield and Nakagawa (2010). Additionally, species name was included as a random factor to control for within‐species non‐independence (i.e. non‐phylogenetic interspecific variation), and also the batch of hormone extraction was included as a random factor to control for inter‐batch variation. On average, the extraction batch accounted for ~10% of the variation in yolk T3 concentration and total contents, and 14% and 18% of the variation in yolk T4 concentration and total contents respectively (Table S3).

Response variables were all assumed to follow a Gaussian distribution. All models were run for 750,000 iterations with a 350 thinning interval and a burn‐in of 50,000, aiming for an effective sample size of approximately 2,000 for all parameters. Following Houslay and Wilson (2017), we used an uninformative parameter‐expanded prior following a Cauchy distribution (*V* = 1, nu = 1, alpha.mu = 0 and alpha.*V* = 25^2^) for all random factors. Using an uninformative inverse‐Wishart prior or improper flat prior gave qualitatively very similar results, suggesting that the results are robust against the choice of priors.

In Bayesian statistics, posterior means of the models for each fixed factor represent an estimate of its correlation with the response variable and its corresponding 95% credible interval (CI) represent the uncertainty around the estimate (Hadfield, 2010). Therefore, 95% CI that did not include 0 gave statistical support for an association or difference (Hadfield, 2010).

#### 2.4.3 Phylogenetic trees and phylogenetic uncertainty

In order to account for phylogenetic uncertainty, each set of models was tested repeatedly across 100 possible trees generated based on two phylogenetic backbones (Ericson et al., 2006; Hackett et al., 2008) and the posterior means of parameter estimates and their 95% CIs were stored (Rubolini et al., 2015). All phylogenetic trees were constructed using BEAST v1.5.1 (Drummond & Rambaut, 2007, for details see Jetz et al., 2012), pruned correspondingly to each phylogenetic set (see Table 1) and obtained from BirdTree.org (Jetz et al., 2012). The results are highly similar across all possible trees (see Figure S5 for examples) and between the two backbones. Therefore, we only report here the results based on the Hackett backbone.

#### 2.4.4 Model diagnostics

Because results were highly similar across all possible phylogenetic trees, we examined model performance by refitting all models with a consensus tree. The consensus tree was derived using the r package phytools (Revell, 2012) for each phylogeny set. Model diagnostics were conducted by visual inspection on the trace plots for proper mixing and on autocorrelations and raised no concern regarding poor mixing or substantial autocorrelation. In support of our inspection, the autocorrelations for all models were <0.05 and Gelman–Rubin diagnostics (Gelman & Rubin, 1992, via the r package coda, Plummer et al., 2006) were all <1.05.

#### 2.4.5 Phylogenetic heritability

We calculated phylogenetic heritability (*H* ^*2*^) as an estimate of phylogenetic signal using the equation:

$$H^{2}=\frac{\sigma_{a}^{2}}{\sigma_{a}^{2}+\sigma_{s}^{2}+\sigma_{e}^{2}},$$

where $\sigma_{a}^{2}$ represents the variance of the phylogeny, $\sigma_{s}^{2}$ represents the variance accounted by individual species (non‐phylogenetic part) and $\sigma_{e}^{2}$ represents the residual variance. The phylogenetic heritability is a measure of the proportion of variance that is explained by phylogeny, conditional on the fixed factors included in the model. Please see Supplementary Methods for more information regarding the properties and interpretation of the phylogenetic heritability.

## 3 RESULTS

### 3.1 Interspecific variation in yolk THs

**FIGURE 1:**
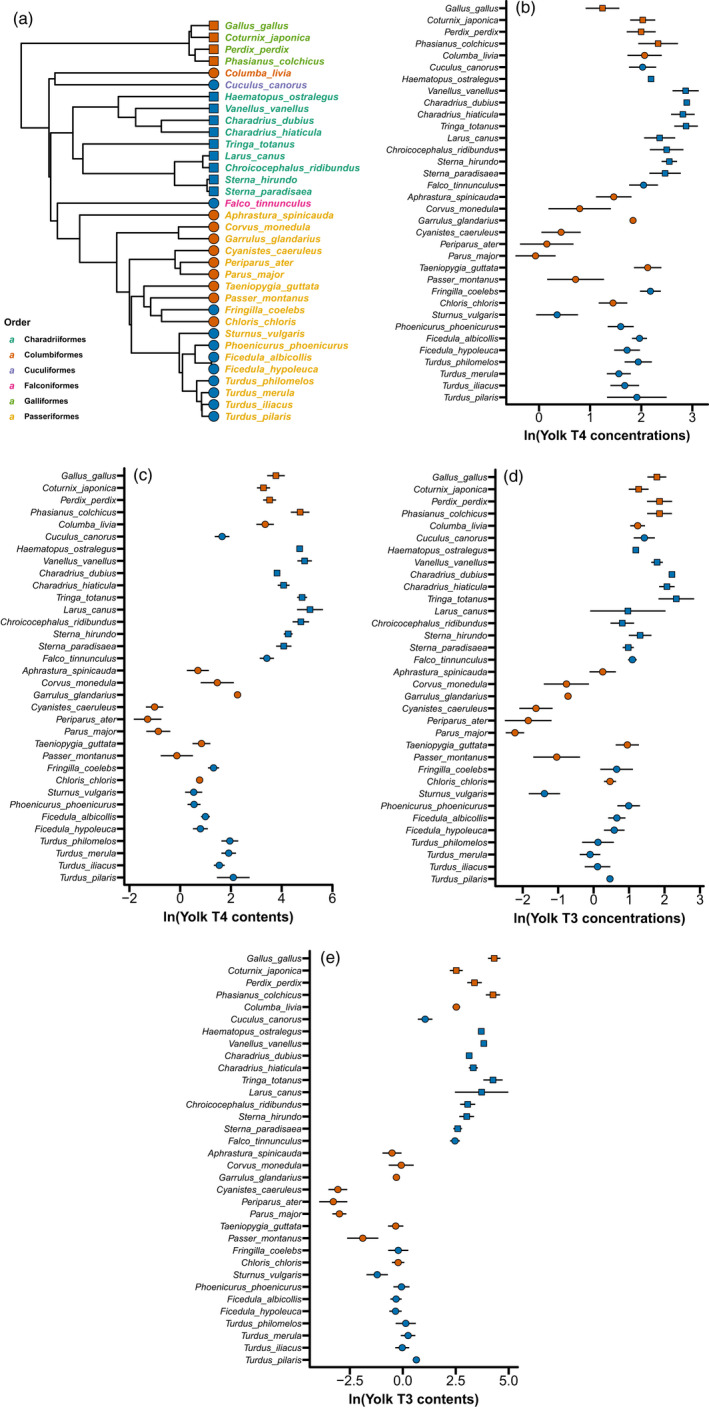
Phylogenetic tree, yolk TH concentrations (pg/mg yolk) and total contents (ng/yolk) of the avian species included in this study. The phylogenetic tree (a) is one possible tree derived from the Hackett backbone (see text). Different colours of species names represent different orders they belong to. The concentrations of yolk T4 (b) and T3 (d) and total contents of yolk T4 (c) and T3 (e) exhibited substantial interspecific variation (mean ± *SD*, see Table S1 for exact values and sample sizes). In all panels, red circles represent resident altricial species, red squares represent resident precocial species, blue circles represent migratory altricial species and blue squares represent migratory precocial species. Silhouettes were obtained from PhyloPic.org.

**FIGURE 2:**
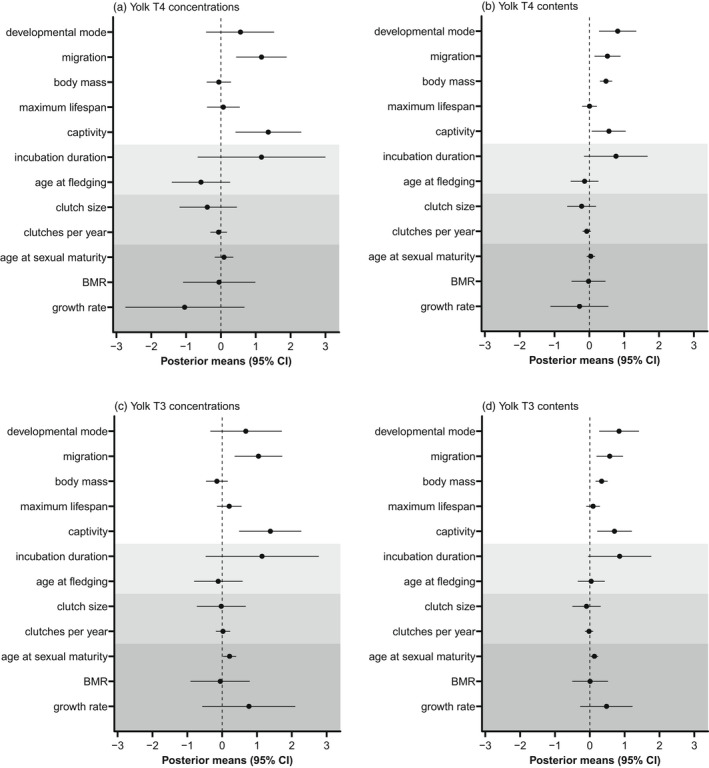
Posterior means (±95% credible intervals) between yolk THs and all life‐history variables tested in this study. The white area presents the estimates from Model set 1 (see text and Table 1). The shaded areas present the variables tested in Model set 2a (the light grey areas), Model set 3 (the medium grey areas) and Model sets 4–6 (the darkest grey area). In Models 2–6, the estimates of the variables that had been tested in Model set 1 are not redundantly presented. For developmental mode, the estimate represents the difference of yolk THs in precocial species from altricial species. For migration, the estimate represents the difference in migratory species from resident species. For captivity, the estimate represents the difference in captive species from wild species.

We observed substantial interspecific variation in both T3 and T4 concentrations (Figure 1; Table S1): the average yolk T3 = 3.15 pg/mg yolk (*SD* = 2.70, CV = 85.71%) and the average yolk T4 = 8.12 pg/mg yolk (*SD* = 4.83, CV = 59.48%). Great tits *Parus major* and redshanks *Tringa totanus* had the lowest and highest concentrations of yolk T3 and T4 among the 34 species respectively. Between the two species, the difference in average yolk TH concentration was 100‐fold for T3 (great tit, mean ± *SD* = 0.112 ± 0.032 pg/mg yolk, *n* = 11; redshank, mean ± *SD* = 11.242 ± 5.067 pg/mg yolk, *n* = 4) and 18‐fold for T4 (great tit, mean ± *SD* = 0.989 ± 0.292 pg/mg yolk, *n* = 12; redshank, mean ± *SD* = 18.101 ± 4.125 pg/mg yolk, *n* = 4). The interspecific variation in total yolk TH contents (T3: mean ± *SD* = 18.06 ± 24.93 ng/yolk, CV = 138.04%; T4: mean ± *SD* = 40.80 ± 51.46 ng/yolk, CV = 106.32%) largely reflected the variation in species body mass, evidenced by the strong correlation between yolk TH contents and species body mass (Figure 2). The lowest total T3 content was observed in the eggs of coal tits *Periparus ater* (*n* = 7, T3: mean ± *SD* = 0.045 ± 0.027 ng/yolk) and the highest in red junglefowl (*n* = 10, mean ± *SD* = 78.122 ± 25.792 ng/yolk). Coal tits also had the lowest T4 contents in the egg yolk (*n* = 7, mean ± *SD* = 0.312 ± 0.157 ng/yolk), whereas common gull (*Larus canus*) had the highest (*n* = 3, mean ± *SD* = 182.853 ± 99.314 ng/yolk).

### 3.2 Phylogenetic heritability

We observed a moderate to strong phylogenetic heritability, indicating that the phylogenetic relatedness among species accounted for 60%–85% of the interspecific variation in yolk THs (Table 2), even after key life‐history traits had been accounted for (see Supplementary methods). On average, T3 had higher phylogenetic heritability than T4, although their 95% CIs largely overlapped with each other's posterior means (Table 2).

**TABLE 2:** Phylogenetic heritability for maternal yolk T3 and T4

Hormone	Parameter	Posterior mean [95% CI]
T3	Concentration	0.84 [0.68, 0.95]
	Total contents	0.84 [0.71, 0.95]
T4	Concentration	0.60 [0.25, 0.87]
	Total contents	0.75 [0.54, 0.91]

### 3.3 Yolk THs and migration

**FIGURE 3:**
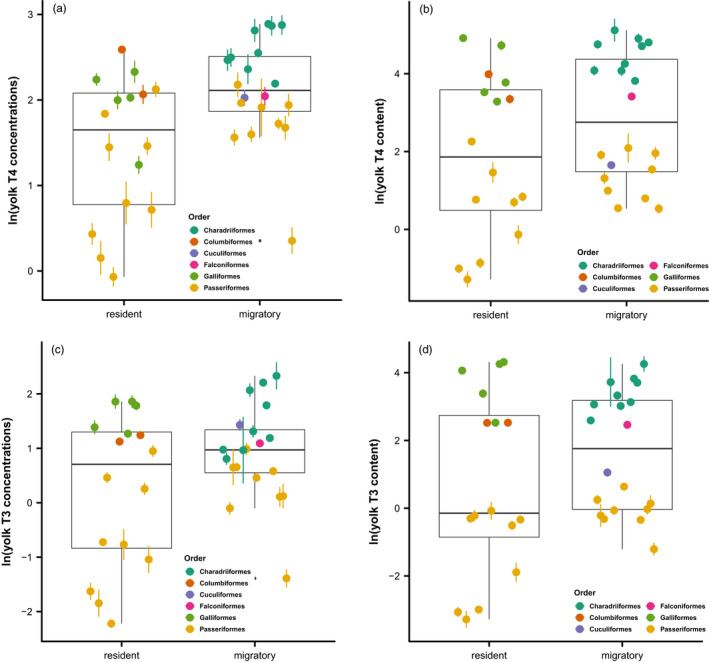
Boxplots and species‐specific averages of yolk TH concentrations (a, c, unit: pg/mg yolk, ln‐transformed) and total contents (b, d, ng/yolk, ln‐transformed) between migratory and resident species. Boxplots represent the median (the middle line) and the first and the third quartiles (the box), and the whiskers extend to 1.5 times of the interquartile range. Coloured dots represent species‐specific means (±*SE*).

Migratory species generally deposited higher concentrations (posterior means and 95% CIs: T3, 1.045 [0.358, 1.726]; T4, 1.166 [0.436, 1.888]) and larger amounts (posterior means and 95% CIs: T3, 0.572 [0.190, 0.954]; T4, 0.519 [0.147, 0.894]) of both THs in the egg yolks than resident species (Figure 3), which was supported by Model set 1 (Figure 2).

### 3.4 Yolk THs and development

**FIGURE 4:**
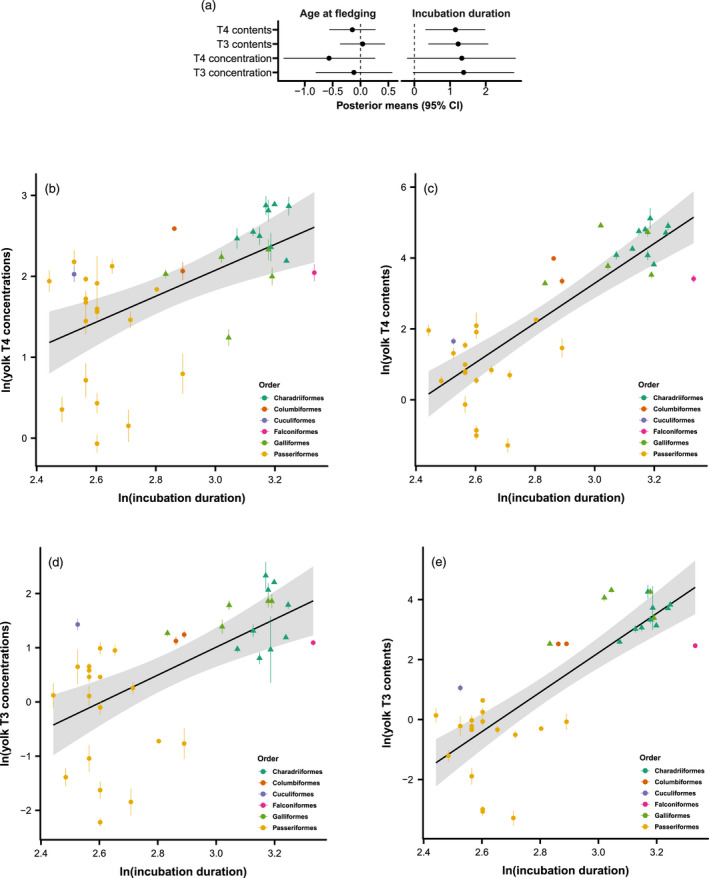
Relationship between developmental duration and yolk THs across species. The phylogenetic mixed model excluding developmental mode suggested that both yolk T4 and T3 contents positively correlated with incubation duration, but not age at fledging (a). This positive correlation between incubation duration and yolk T4 (c) and T3 contents (e) was clear when plotting the raw data (species‐specific means ± *SE*). Yolk T4 and T3 concentrations also exhibit positive but weaker correlations with incubation duration (b, d), as reflected by the wider associated credible intervals (a). The concentrations (b, d, unit: pg/mg yolk) and total contents (c, e, unit: ng/yolk) were both ln‐transformed. Dots and triangles represent altricial and precocial species respectively. Black lines (shaded areas: 95% CI) represent the average correlation between incubation duration and yolk THs across all species.

Precocial species deposited larger total amounts of THs (both T3 and T4) in the yolks than altricial species (Figure 4), but not higher TH concentrations. Model set 1 supported this difference with positive posterior means and 95% CIs for estimates on TH contents (T3, 0.841 [0.270, 1.412]; T4, 0.814 [0.278, 1.348], Figure 2b,d) but not concentration (T3, 0.680 [−0.337, 1.714]; T4, 0.562 [−0.426, 1.527], Figure 2a,c), when controlling for species body mass and other life‐history traits. Neither incubation duration nor age at fledging were associated with yolk THs (Model set 2a, Figure 2; Table S4). However, when developmental mode was removed from the model (i.e. Model set 2b), the total amounts of both yolk T3 and T4 were positively correlated with incubation duration but not with age at fledging (posterior means and 95% CIs: T3, 1.227 [0.391, 2.068]; T4, 1.151 [0.309, 1.987], Figure 4a). The concentrations of yolk T3 and T4 also showed positive correlations with incubation duration, but the 95% CIs slightly overlapped 0 (posterior means and 95% CIs: T3, 1.380 [−0.039, 2.796]; T4, 1.329 [−0.207, 2.838], Figure 4a,b,d).

### 3.5 Yolk THs and pace‐of‐life‐related traits

Species body mass strongly correlated with yolk TH contents, but not with yolk TH concentration (Figure 2). Age at sexual maturity positively correlated with yolk T3 concentration (posterior mean and 95% CI = 0.214 [0.028, 0.403], Figure 2c) and total contents (posterior mean and 95% CI = 0.132 [0.027, 0.237], Figure 2d), but not with yolk T4 (Table S4; Figure S6). No other pace‐of‐life‐related trait, including fecundity, maximum life span, BMR and growth rate, was correlated with maternal yolk TH concentrations or contents in Model sets 3–6 (Figure 2). Given the previous findings that migratory birds have higher BMR (Jetz et al., 2008) and live faster (Soriano‐Redondo et al., 2020), we additionally repeated the models excluding migratory status to examine whether pace‐of‐life‐related traits would show associations with yolk THs. However, no clear associations with yolk THs were found in these models.

### 3.6 Yolk THs and captivity

Our models consistently suggested that captive species deposited higher concentrations and larger total amounts of both yolk T3 and T4 than wild species (Figure 2; Figure S7; Table S4).

## 4 DISCUSSION

Our phylogenetic mixed models suggest clear differences in maternal yolk THs between migratory and resident species and between precocial and altricial developmental modes. However, contrary to our expectation, we found no statistical support for associations between maternal yolk THs and pace‐of‐life‐related traits, after controlling for body mass. These results suggest that among the life‐history traits we tested, the associations with THs are more likely related to migration (Hypothesis 1) and developmental mode (Hypothesis 2), but not pace‐of‐life (Hypothesis 3). Moreover, captive life appears to exert some influence on maternal TH transfer, probably reflecting phenotypic plasticity, physiological acclimation or some unintentional selective force due to the captive environment.

### 4.1 Interspecific variation and phylogenetic heritability of yolk THs

For the first time, we assessed the interspecific variation in maternal yolk THs in birds. Passerines, particularly tits (family: Paridae), tended to have the lowest TH levels in egg yolks, whereas waders (order: Charadriiformes) tended to have the highest yolk TH levels. The variation across species is large (CV of yolk TH concentrations = 85.71% and 59.48% for T3 and T4, respectively), but comparable to other yolk hormones [e.g. yolk testosterone in birds ranges from 2.37 to 51.10 pg/mg yolk (mean ± *SD* = 10.32 ± 10.12, *n* = 101 species), with a CV = 98.06%, and yolk androstenedione ranges from 2.54 to 213.95 pg/mg yolk (mean ± *SD* = 34.51 ± 38.97), with a CV = 112.92% (Gil et al., 2007)].

Even after taking into account key life‐history traits (i.e. migratory status, developmental mode and body mass) and the influence of captivity, we still detected moderate to strong phylogenetic heritability on both yolk THs. This indicates that besides life histories, a substantial proportion of variation in maternal THs is accounted for by the phylogeny itself. Our assessment on raw yolk TH data also indicated that the interspecific variation of yolk THs conforms to the stochastic evolutionary process predicted by Brownian motion based on the values of Pagel's *λ*. While the phylogenetic dependence of yolk THs could arise from a conserved physiological mechanism underlying maternal TH transfer to egg yolks, it seems premature to discuss about the processes underlying the observed phylogenetic signal, given our sample size and the predominance of passerines in our sampling.

### 4.2 THs and migration

Our results supported the hypothesis that migratory life history is associated with higher maternal TH transfer in egg yolks. Higher yolk THs might mirror elevated circulating THs in migratory species, which might be directly or indirectly linked to their higher BMRs (Jetz et al., 2008). In line with this explanation, in two closely related skylark species, the migratory Eurasian skylarks *Alauda arvensis* had higher pre‐breeding blood T3 levels than the resident Asian short‐toed larks *Calandrella cheleensis* (Zhao et al., 2017). In fish, a marine migratory ecotype of the stickleback *Gasterosteus aculeatus* also had higher plasma T4 than a stream resident ecotype (Kitano et al., 2010).

The difference between migratory and resident species could also be due to temporal changes in THs. Given the crucial regulatory role of THs on migration (e.g. Pant & Chandola‐Saklani, 1993; Pérez et al., 2016), one plausible scenario could be that circulating THs were elevated during migration and had not returned to the non‐migrating level during egg formation, which occurs right after vernal migration in migratory species, while resident species lack such seasonal variation in circulating THs. In migratory red knots *Calidris canutus*, high plasma T4 coincided with the period of high body mass increase in spring (Jenni‐Eiermann et al., 2002), consistent with the proposed function that T4 stimulates fattening and muscle hypertrophy in preparation of migration (Pérez et al., 2016). In migratory brent geese *Branta bernicla*, plasma T3 was also found to be higher in spring than in the wintering grounds (Poisbleau et al., 2006). In comparison, in resident great tits and willow tits *Poecile montanus*, an increase of T4 was observed only during the post‐breeding moult period and not during the pre‐breeding stage (Silverin et al., 1989). These studies support the idea that migratory birds have different temporal variation in blood THs compared with resident species. However, we still need comprehensive phylogenetic comparative studies to verify whether migratory and resident species differ in their circulating THs across seasons.

At the intraspecific level, there is variation in whether individuals are migratory or resident, which can depend on local conditions (Acker et al., 2021; Chapman et al., 2011; Reid et al., 2018). The physiological mechanism underlying such variation in migration propensity is unclear although various perspectives have been proposed (Hegemann et al., 2019). Based on our findings, it is possible that maternal yolk THs might predispose an individual to migration via the activational or organizational effects, which would be an interesting topic for future investigation.

### 4.3 Does precocial development require more maternal THs?

Our finding that precocial species deposited larger amounts of THs in egg yolks than altricial species (while accounting for body mass) fits with their distinct developmental trajectories (Starck & Ricklefs, 1998). Chicks of precocial species hatch at a more advanced developmental stage in terms of visual and locomotory ability (Starck & Ricklefs, 1998), endocrine function (de Groef et al., 2013; McNabb, 2006; Wada, 2008) and thermoregulation (Price & Dzialowski, 2018). Our result therefore suggests that the larger amount of maternal THs in eggs of precocial species may be related to this advanced ontogenetic trajectory. However, it is currently too early to tell whether the larger amounts of yolk THs may be a cause of precocial development or a consequence.

The larger amounts of yolk THs in precocial species is after body mass is controlled for in our model and does not reflect an allometric relationship between yolk THs and body mass. Developmental mode therefore explains some variation in yolk THs that is not predicted by body mass. An interesting point here is that precocial species also have a higher yolk proportion in the egg than altricial species (Collins & LeCroy, 1972; Ricklefs, 1977). This can explain the larger amounts of yolk THs in precocial species and also might be a hint that the maternal TH transfer is linked with the process of vitellogenin (yolk precursor) accumulation during egg formation. Further studies to examine the covariation between yolk mass and yolk THs as well as experiments to test the role of vitellogenin in maternal TH transfer will provide further insights into these questions.

Although precocial species have both longer prenatal and postnatal developmental periods than altricial species (Figure S4), our models suggested that maternal THs are only positively correlated with incubation duration, but not age at fledging, when the model did not control for developmental mode (Figure 4). This finding is consistent with the major functions maternal THs have on embryonic development. One possible interpretation of this result could be that larger amounts of maternal yolk THs extend the prenatal developmental period, hence allowing precocial chicks to hatch at a more advanced stage. Nevertheless, such an interpretation contradicts the results of within‐species studies. In experiments with a direct elevation of yolk THs in altricial pigeons and precocial quail, incubation duration was not extended (Hsu et al., 2017; Sarraude et al., 2020b). In fact, experimentally elevated yolk THs might even shorten incubation duration (in great tits, Cossin‐Sevrin et al., 2022). In a reptile species (short‐necked turtles *Emydura macquarii*), exogenously injected T3 in the middle of incubation was also found to shorten incubation duration (McGlashan et al., 2017). Therefore, either the relationship between yolk THs and prenatal development are opposite at the inter‐ versus intraspecific level, or the positive association between yolk THs and incubation period across species may not be causal.

### 4.4 No size‐independent associations between maternal THs and pace‐of‐life traits

Despite the known function of THs in metabolism, we did not detect associations between maternal yolk THs and most pace‐of‐life‐related traits. The only finding was a weak positive correlation suggesting that species that become sexually mature at an older age (i.e. having a slower pace‐of‐life) deposit higher levels of T3, but not T4, in egg yolks. This result contradicts our expectation that higher T3 being associated with a ‘faster’ pace‐of‐life, that is earlier sexual maturation. Importantly, no other pace‐of‐life trait showed a similar trend. We therefore do not consider that this result alone supports our hypothesis that maternal TH transfer covaries with pace‐of‐life syndrome.

The lack of associations with most of the pace‐of‐life‐related traits could have several non‐mutually exclusive explanations: First, our hypothesis for THs to be involved in pace‐of‐life is based on the interactions between THs and BMR, as well as with glucocorticoids. However, while the pace‐of‐life framework suggests a ‘nexus’ of physiological and life‐history traits (Ricklefs & Wikelski, 2002), this does not necessarily pose a constraint that forces related physiological systems to exhibit correlated phenotype (Versteegh et al., 2012). Therefore, THs and pace‐of‐life‐related traits might display complex relationships that are difficult to characterize (cf. glucocorticoids, Crespi et al., 2013). Second, some constraints in our methods may have limited our power to detect the relationship between THs and pace‐of‐life‐related traits. Our sampling mostly focused on temperate species for logistic reasons, and it is thus possible that our sampling did not capture enough variation across the pace‐of‐life syndrome. Future studies should therefore consider expanding the sampling to a more comprehensive list of species across a larger geographical scale if possible. Alternatively, focusing on a smaller clade that is distributed across a wider latitudinal range could be promising, such as the stonechat (*Saxicola spp*.) model system that shows variation across the pace‐of‐life syndrome (Wikelski et al., 2003). Third, we have to keep in mind that what we examined in the present study are THs transferred from the mother to egg yolks, not THs in blood circulation. Although some studies have shown parallel changes in yolk THs in response to experimentally manipulated circulating THs (Sarraude et al., 2020c, 2021; Wilson & McNabb, 1997), circulating THs may not explain all variation observed in yolk THs (e.g. Hsu et al., 2016). Therefore, our result suggests that yolk THs do not show associations along the pace‐of‐life continuum but we cannot exclude the possibility that variation in blood THs might still align with pace‐of‐life trait variation. Further comparative studies on blood circulating THs are therefore needed to answer this question.

### 4.5 Captive life is associated with higher maternal TH deposition

Our models consistently suggested that captive species deposited higher concentrations and larger total amounts of THs in the egg yolks than wild species. Although we did not have a specific hypothesis on how captivity would be correlated with yolk THs, a straightforward explanation to this result lies in the differences between captive and wild environment, including food availability and chronic stress (Beaulieu, 2016; Mason, 2010). A number of studies have reported cases in which captive animals exhibit different morphology, physiology, behaviour and genetic variation from their free‐living conspecifics (e.g. Beaulieu, 2016; Forstmeier et al., 2007; Gilby et al., 2013; Romero & Wingfield, 1999). Therefore, the higher deposition of THs in egg yolks by captive species likely reflects phenotypic plasticity or intentional (i.e. selective breeding) or unintentional selection. Indeed, there were several domesticated species among the captive species in our dataset, which may have driven this result. The actual underlying factors are elusive at the moment, but likely to be manifold. A direct comparison of conspecific captive and wild populations would be a fruitful next step to understand the consequences of captivity on yolk TH deposition and identify potential causal factors.

### 4.6 Study limitations

Our study revealed intriguing patterns, but our relatively low sample size (*n* = 34 species) and large proportion of passerines (*n* = 18 species) warrants some caution. Errors and uncertainty in life‐history trait values and hormone data, partly because of the small within‐species sample sizes in some species, are also relevant when interpreting the results. Nevertheless, the identified patterns we report here suggest that aspects of life‐history variation are associated with maternal TH transfer into egg yolks.

## 5 CONCLUSIONS

Our study reveals large variation in egg THs across bird species. Phylogenetic mixed models revealed that a part of this inter‐species variation is explained by migratory status and developmental mode, with migratory species depositing higher concentration and total amounts of THs in egg yolks than resident species and precocial species, which have longer prenatal development, depositing larger total amounts of THs in egg yolks than altricial species. Intriguingly, we did not find evidence that maternal THs were related to pace‐of‐life‐associated traits. The strong phylogenetic heritability implies that the physiological mechanism responsible for maternal TH transfer may be highly conserved across species. Further effort should be invested to uncover the physiological mechanisms underlying the observed patterns of interspecific maternal TH transfer as well as variation in adult circulating THs. Interspecific studies on maternally transferred THs, like this one, will also benefit from a larger sample size across a greater phylogenetic range and geological scale. Since variation in migration and developmental mode are not unique to birds, whether maternal THs may associate with life‐history variation also in other vertebrate taxa awaits further investigation.

## CONFLICT OF INTEREST

The authors claim no conflict of interest.

## AUTHORS' CONTRIBUTIONS

S.R. conceived and coordinated the study and conducted hormone analysis; B.Y.H. conducted all statistical analysis and drafted the manuscript. All authors contributed samples for this study and made substantial contribution to the revisions of the manuscript.

## Supporting information


Supinfo S1

Supinfo S2

## Data Availability

The data and the R code used to produce the results of this study are available from the Dryad Digital Repository https://doi.org/10.5061/dryad.547d7wmb5 (Hsu et al., 2022).
