# Supplementary material for: Maternally transferred thyroid hormones and life‐history variation in birds
Source: J Anim Ecol. 2022 May 7;91(7):1489–506. doi: 10.1111/1365-2656.13708 (PMC9546341; doi:10.1111/1365-2656.13708)
Supplement: Supplementary file 2 — Supinfo S2 [file JANE-91-1489-s002.pdf]

Supplementary methods and results for

Maternally-transferred thyroid hormones and life-history variation in birds

Bin-Yan Hsu[1*], Veli-Matti Pakanen[2,3], Winnie Boner[4†], Blandine Doligez[5†], Tapio Eeva[1†], Ton G.G. Groothuis[6†], Erkki Korpimäki[1†], Toni Laaksonen[1†], Asmoro Lelono[6, 7†], Pat Monaghan[4†], Tom Sarraude[1,6†], Robert L. Thomson[8†], Jere Tolvanen[2†], Barbara Tschirren[9†], Rodrigo A. Vásquez[10†], Suvi Ruuskanen[1,11]

[1] Department of Biology, University of Turku, Finland

[2] Ecology and Genetics Research Unit, University of Oulu, Finland

[3] Department of Biological and Environmental Sciences, University of Gothenburg, Sweden

[4] Institute of Biodiversity, Animal Healthy and Comparative Medicine, University of Glasgow, Scotland, United Kingdom

[5] Department of Biometry and Evolutionary Biology, CNRS UMR 5558, Université de Lyon 1, France

[6] Groningen Institute for Evolutionary Life Sciences (GELIFES), University of Groningen, the Netherlands

[7] Biology Department, Natural Sciences and Mathematics Faculty, Jember University of Indonesia, Indonesia.

[8] Fitzpatrick Institute of African Ornithology, DST-NRF Centre of Excellence, University of Cape Town, South Africa

[9] Centre for Ecology and Conservation, University of Exeter, United Kingdom

[10] Instituto de Ecología y Biodiversidad, Departamento de Ciencias Ecológicas, Facultad de Ciencias, Universidad de Chile, Santiago, Chile

[11] Department of Biological and Environmental Sciences, University of Jyväskylä, Finland

* Corresponding author: biyahs@utu.fi, TEL: +358294504250, Address: Turun Yliopisto, FI-20014, Turku, Finland

† These authors are listed in alphabetical order.

Supplementary methods

Yolk TH analysis

Before TH extraction, all eggs were first dissected to separate yolk from albumen after a short period of thawing. A few eggs showed signs of very early development and all such eggs were discarded from the analysis. After dissection, we first weighed the whole yolks (~0.01 g), and then took and weighed a half (for passerines) or a quarter (for other species) of the yolk for homogenization. Because yolk hormones do not evenly distribute across yolk layers (Lipar et al. 1999; Hackl et al. 2003), using either a half or a quarter of the yolk ensured the hormones quantified will be representative of the whole yolk. We then added MQ water in an amount approximately equal to the mass of yolk samples to facilitate homogenizing. The amount of added MQ, as long as recorded and factored in during the calculation process, does not influence or bias the extraction and quantification process of yolk THs. We then added a few metal beans to the yolk-MQ mixture and placed it on a tissue lyser (Qiagen) for 3 minutes for homogenization.

After homogenization, we took a subsample (ca. 300 mg) of the yolk-MQ mixture for TH extraction. The extraction protocol we used has been described previously (Ruuskanen et al. 2016a). We first added 2 ml of methanol to the yolk-MQ mixture and well vortexed the sample. During the analysis of hormones, usually there is no guarantee that 100% of the targeted hormone in the sample will be measured because hormones might only be partly extracted, or get lost during the extraction or purification process. Ignoring this may largely underestimate the true amount of hormones present in the samples. Therefore, we added a known amount of $^{13}C_{12}$-T4 (Larodan) to each sample as an internal tracer, which allowed us to estimate the percentage of hormones that has been successfully extracted and measured. This percentage represents the “extraction efficiency” (commonly called “recovery”) and correcting for it is crucial for getting more accurate measurements.

Next, 4 ml of chloroform was then added and samples were centrifuged at +4°C 1900g for 15 min. The supernatant was then collected and the pellet was re-extracted in 2:1 chloroform-methanol

mixture, back-extracted with 0.05% $CaCl_2$ into aqueous phase, and re-extracted with chloroform:methanol:0.05% $CaCl_2$ mixture (3:49:48). The aqueous phase was further purified on Bio-Rad AG 1-X2 resin columns, eluted with 70% acetic acid, and then vacuum-evaporated overnight. Samples from different species were spread over extraction batches and the extraction batch ID was included as a random intercept in the statistical models to account for between-batch variation.

We used a nano-flow liquid chromatography-mass spectrometry (LC-MS) protocol that has good repeatability (within-sample CV < 10%, Ruuskanen et al. 2018) and has been validated for several Galliform and Passeriform species of birds previously (Ruuskanen et al. 2018) to measure the total (i.e. free unbound hormones + bound hormones) yolk T3 and T4 simultaneously. In brief, the dry TH extracts were re-suspended in 150 µl 0.1% NH_3 and further diluted with 0.01% NH_3. The dilution factor depended on the expected amount of T3 and T4 in each sample. Internal standards $^{13}C_6$-T3 and $^{13}C_6$-T4 were added to identify and also quantify T3 and T4 in the samples. A triple quadruple mass spectrometer (TSQ Vantage, Thermo Scientific, San Jose, CA) and nano-flow HPLC system Easy-nLC (Thermo Scientific) were used for the measurements. The on-column quantification limits were 10.6 amol and 17.9 amol for T4 and T3, respectively (Ruuskanen et al. 2018). The data was acquired automatically using Thermo Xcalibur software (Thermo Fisher Scientific) and subsequently analysed by Skyline (MacLean et al. 2010). TH concentrations were calculated using the peak area ratios of sample to internal standard, calibrated by a standard line ($R^2 \geq 0.99$), corrected for recovery, and expressed as pg/mg yolk. We further calculated the total amount of THs per yolk by multiplying the measured concentrations with the yolk mass and expressed it as ng/yolk.

Across all samples we measured, the average recovery varied from 30.90% to 52.01% (i.e. between-species CV of recovery = 12.95%) and there appeared no clear pattern of systematic bias across species (Fig. S3). The typical recovery for THs is 40-60% for T4 (van Herck et al. 2012). Our recovery is therefore on the lower bound but not uncommon. The correction for recovery is therefore warranted to avoid substantial underestimation. Within each species, recovery also varied

across individuals. The within-species CV of extraction efficiency varied from 14.06% in chaffinch (*Fringilla coelebs*) samples to 53.94% in blue tit (*Cyanistes caeruleus*) samples.

Sample storage and assessment of storage effects

All collected eggs were frozen and stored at -20°C on the day of collection and for most of the species, the frozen eggs were shipped to the University of Turku, where we separated the yolk for hormone analysis in 2017 (see *Yolk TH analysis*). Two exceptions are the collared flycatchers (collected in 2011) and the rock pigeons (collected in 2014), for which the eggs had been dissected and the yolk had been homogenized and mixed in known amount of milli-Q (MQ) water and stored at -80°C for long-term storage before shipped to University of Turku and were already ready for extraction.

In order to examine whether storage duration may have influenced yolk THs (e.g. due to degradation over time), we compared yolk T3 and T4 concentrations from the eggs collected in 2016 (n=122) and 2017 (n=48) and controlled for species differences (as a random factor, non-phylogenetically). The results indicated no clear decrease in yolk TH concentrations due to longer storage (i.e. eggs from 2016 compared to 2017): There was no difference in the average T3 between the two years (LMM, t=0.595, p=0.554, Fig. S2 left panel); yolk T4 concentrations in eggs from 2017 were on average non-significantly lower than those in eggs from 2016 (LMM, t=-1.881, p=0.063, Fig. S2 right panel), which cannot be attributed to storage effects as degradation over time should only decrease TH levels after longer storage (i.e. eggs from 2017 should show higher levels of THs instead of lower). Since the year difference was not significant, we did not include year in the model for the sake of simplicity.

As our data include eggs under longer storage (eggs of collared flycatchers and rock pigeons), we also explored the potential storage effects more thoroughly by examining our previously published data (Ruuskanen et al. 2016b) in great tits (*Parus major*), in which eggs (total n=188, n per year varies from 19 to 49) were collected in 2006-2010 and stored at -80˚C for years

until they were analysed in 2013 (i.e. storage time 3-7 years). Our examination of yolk T3 and T4 concentrations showed that among the eggs stored for 3-5 years, some have high levels of T3. However, such high values are absent among the eggs stored for 6 and 7 years (Fig. S3 left panel). Statistically, this led to a weak decreasing pattern in yolk T3 concentrations over the storage duration (range: 3-7 years, GLM, $r=-0.016\pm0.007$ on ln scale, $p=0.023$). No such pattern was observed for yolk T4 ($r=0.013\pm0.025$, $p=0.598$, Fig. S3 right panel). Therefore, although it is not possible to tell the true cause of this pattern, one possibility is that some T3 have degraded over 5 years of storage. In other words, this effect does not appear to occur at < 5 years of storage.

In this study, the eggs of most species were collected in 2016 and 2017. The only three exceptions are the eggs of junglefowl (collected in 2015), rock pigeons (collected in 2014), and collared flycatchers (collected in 2011). Therefore, the only data that may have been influenced by such a presumed storage effect is limited in one species, the collared flycatcher. Nevertheless, because yolk T3 concentrations in collared flycatchers did not exhibit observable anomalies compared to yolk T3 in its sister species, the pied flycatchers (*F. hypoleuca*) or among all passerine species (see Fig. 1 in the main paper), we think that even in the case of a storage degradation, it should only induce potential bias in this single species and is unlikely to bias our results.

Additional information on the collection of life-history traits

I. Developmental mode and developmental durations

We specified each species as precocial or altricial, based on the description in Cramp (1977-1994) and following the principle and criteria in Starck and Ricklefs (1998) and determined the values of incubation duration and age at fledging from Myhrvold et al. (2015). We define “fledging” as when altricial nestlings leave the nest and when precocial chicks are able to fly, following Myhrvold et al. (2015). In birds, incubation duration and age at fledging are commonly used to represent the length of the prenatal and postnatal developmental period (e.g. Gorman and Williams 2005; Gil et al. 2007; Schwabl et al. 2007).

II. Mass-specific basal metabolic rates (BMR)

BMR data were compiled from the sources documented in the *Supplementary data*. All data were first converted to KJ/h and divided by the body mass recorded in each respective literature to calculate the mass-specific BMR (KJ/h/g).

Whole-body BMR has an exponential relationship with body mass (Nagy et al. 1999), which becomes linear on a logarithmic scale:

$$BMR = \mathrm{a} \times \mathrm{mass}^{b}$$

$$log(BMR) = \log(\mathrm{a}) + \mathrm{b} \times \log(mass)$$

When dividing by mass, the so-called "mass-specific BMR" still has a linear relationship with body mass:

$$BMR/mass = \mathrm{a} \times \mathrm{mass}^{b-1}$$

$$log(Masss\text{-}pecific\ BMR) = \log(\mathrm{a}) + (\mathrm{b} - 1) \times \log(mass)$$

Because the scaling coefficient b is usually less than 1 in vertebrates (Nagy et al. 1999, 0.6-0.8 in birds depending on species, McKechnie et al. 2006, McNab 2009), the relationship between mass-specific BMR and species' body mass is negative. We therefore further corrected the mass-specific BMR data for body mass and phylogeny (see *Model specifications* in the main text).

III. Maximum lifespan

The data of maximum lifespan and ages at sexual maturity almost exclusively originated from the database AnAge (Tacutu et al. 2013), except for the thorn-tailed rayaditos (*Aphrastura spinicauda*), for which the data was obtained from Moreno et al. (2005) and Quirici et al. (2019). The AnAge database compiles data from various sources and therefore the data quality is heterogeneous. The data of maximum lifespan likely comes from captive individuals for many species, which may be related to certain life histories. Nevertheless, maximum lifespan is mostly determined by intrinsic factors and less sensitive to extrinsic cause of death, such as predation (Barja

2013). Therefore, some have argued that maximum lifespan could represent the true potential of longevity of a species better (Barja 2013, Vágási et al. 2019). Moreover, the availability of the data of maximum lifespan is far better than other measures of longevity, such as median lifespan (de Magalhães et al. 2007; Healy et al. 2014). In fact, maximum lifespan has been widely used in comparative studies of aging (e.g. Tricola et al. 2018).

Phylogenetic heritability

As described in the text, we calculated phylogenetic heritability (H^2) as a measure of phylogenetic signal:

$$H^2 = \frac{\sigma_a^2}{\sigma_a^2 + \sigma_s^2 + \sigma_e^2}$$

where σ_a^2 represents the variance of the phylogeny; σ_s^2 represents the variance accounted by individual species (non-phylogenetic part); σ_e^2 represents the residual variance.

Because phylogenetic heritability is in fact the proportion of the phylogenetic variance over the total phenotypic variance, the fixed factors included in the model will influence the value of the phylogenetic heritability by changing the estimates of all variance components (Nakagawa and Schielzeth 2010). Therefore, we calculated the phylogenetic heritability for yolk T4 and T3 based on the phylogenetic mixed model that only included the life-history traits whose 95% CIs did not encompass zero. These are developmental mode, migratory status, captivity, and body mass (see *Results*). In these models, all 34 species and the full phylogeny set was included and we used a consensus tree from the Hackett backbone (Hackett et al. 2008), derived by using the *phytools* package (Revell 2012).

Although the H^2 is mathematically equivalent to Pagel's λ (Hadfield and Nakagawa 2010), our estimates of H^2 in Table 2 are substantially lower than the estimated Pagel's λ based on the species average yolk T3 and T4 using the package *geiger* (close to 1, see *Brownian motion versus Ornstein-Uhlenbeck process*). Such discrepancy may be explained by how the variation in yolk T3 and T4 was

partitioned by the two methods. In addition to phylogeny, the values of yolk T3 and T4 have many other sources of variation, including the true biological variation within each species (e.g. Hsu et al. 2019) and measurement errors, which should be properly controlled for (Ives et al. 2007). To this end, the *fitContinuous* function (in the package *geiger*) uses the standard error of the mean and does not allow other explanatory factors to be considered. In contrast, the phylogenetic mixed model has a much higher flexibility to account for and partition different sources of variation. Specifically, the phylogenetic mixed models allow us to partition the non-phylogenetic interspecific variation (σ_s^2), and therefore reduced the variation that was attributed to phylogeny. Moreover, fixed factors always control for some variance in the response variable, thus decreasing the variance left for random effects (and residual). As our fixed effects were life-history traits that generally tend to co-vary with the phylogeny, they may have removed some of the σ_a^2, compared to if less or no fixed effects were fitted. This would also result in reduced estimates of phylogenetic heritability. To illustrate this point, we calculated the phylogenetic heritability from the models described above but removed captivity, and also removed all fixed factors. We listed all resultant estimates of H^2 in Table S4 to compare with our reported H^2. The comparison clearly shows that the fewer fixed factors included, the higher the estimates of H^2. Therefore, our reported H^2 should be interpreted as the proportion of the “remaining” variance that is explained by phylogeny, after considering all relevant fixed factors.

Between-batch variation of yolk THs

The proportion of variance explained by the extraction batch was calculated as

$$\frac{\sigma_b^2}{\sigma_a^2 + \sigma_s^2 + \sigma_b^2 + \sigma_e^2}$$

where σ_b^2 represents the estimated variance of the extraction batch. The denominator is the summation of variance of all random factors and the residual variance.

Supplementary tables and figures

Table S1. Mean ± SD of yolk T3 and T4 concentrations and total contents of all 34 species in this study.

Species	N of T3	Yolk T3 concentration (pg/mg yolk)	Yolk T3 content (ng/yolk)	N of T4	Yolk T4 concentration (pg/mg yolk)	Yolk T4 content (ng/yolk)
**Japanese quail, *Coturnix japonica***	21	3.690±1.009	13.035±3.787	21	7.802±1.661	27.514±6.846
**Red jungle fowl, *Gallus gallus gallus***	10	6.163±1.852	78.122±25.792	10	3.633±1.215	45.991±16.501
**Domesticated chicken, *Gallus gallus domesticus***	5	4.151±1.315	59.809±16.053	5	9.488±1.621	137.149±16.649
**Grey partridge, *Perdix perdix***	7	6.803±2.765	31.229±12.491	7	7.633±2.159	34.930±9.072
**Ringed-necked pheasant, *Phasianus colchicus***	9	6.743±2.010	74.055±22.609	9	11.060±5.022	120.725±52.997
**Common cuckoo, *Cuculus canorus***	8	4.322±1.065	2.998±0.788	8	7.821±2.112	5.397±1.462
**Rock pigeon, *Columba livia livia***	9	3.532±0.785	12.685±2.351	9	8.303±2.982	29.978±10.289
**Homing pigeon, *Columba livia domesticus***	4	3.103±0.463	12.575±2.084	4	13.339±0.687	53.981±3.341
**Eurasian oystercatcher, *Haematopus ostralegus***	1	3.285	40.782	1	8.946	111.068
**Northern Lapwing, *Vanellus vanellus***	5	6.051±0.942	46.195±6.027	5	18.050±4.497	139.166±39.438
**Little ringed plover, *Charadrius dubius***	1	9.098	22.972	1	17.984	45.409
**Common ringed plover, *Charadrius hiaticula***	3	8.021±1.630	28.325±5.727	3	16.965±3.871	59.980±14.134
**Redshank, *Tringa tetanus***	4	11.242±5.067	76.245±32.722	4	18.101±4.125	123.911±25.503
**Common tern, *Sterna hirundo***	8	3.883±1.250	21.563±7.313	8	12.920±1.844	71.466±11.647
**Arctic tern, *Sterna paradisaea***	6	2.681±0.429	13.602±2.926	6	12.229±3.479	61.544±17.678
**Common gull, *Larus canus***	3	3.723±3.521	67.738±75.481	3	10.929±3.444	182.853±99.314
**Black-headed gull, *Chroicocephalus ribidundus***	9	2.363±0.883	22.773±9.292	9	12.789±4.801	121.907±43.035
**Kestrel, *Falco tinnunculus***	7	3.000±0.356	12.001±2.698	7	7.992±2.238	31.472±9.262
**Thorn-tailed rayadito, *Aphrastura spinicauda***	11	1.375±0.503	0.656±0.278	11	4.564±1.647	2.192±0.996
**Jackdaw, *Corvus monedula***	5	0.552±0.390	1.079±0.703	6	2.529±1.248	4.982±2.464
**Eurasian jay, *Garrulus glandarius***	1	0.485	0.739	1	6.287	9.568
**Blue tit, *Cyanistes caeruleus***	9	0.217±0.104	0.051±0.024	9	1.647±0.659	0.385±0.134
**Great tit, *Parus major***	11	0.112±0.032	0.053±0.020	12	0.989±0.292	0.458±0.162

**Coal tit,** ***Periparus ater***	7	0.186±0.106	0.045±0.027	7	1.301±0.619	0.312±0.157
**Zebra finch,** ***Taeniopygia guttata***	9	2.729±1.084	0.767±0.372	9	8.681±2.733	2.462±1.028
**Tree sparrow,** ***Passer montanus***	7	0.415±0.232	0.187±0.127	7	2.294±1.046	1.015±0.510
**Chaffinch,** ***Fringilla coelebs***	2	2.016±0.896	0.853±0.391	2	8.927±1.798	3.768±0.815
**Greenfinch,** ***Chloris chloris***	3	1.603±0.285	0.826±0.244	3	4.366±1.276	2.160±0.273
**European starling,** ***Sturnus vulgaris***	7	0.273±0.131	0.334±0.179	7	1.537±0.695	1.794±0.670
**Common redstart,** ***Phoenicurus phoenicurus***	8	2.805±0.850	0.998±0.367	8	5.073±1.151	1.774±0.424
**Collared flycatcher,** ***Ficedula albicollis***	15	1.978±0.489	0.753±0.201	15	7.209±0.998	2.737±0.418
**Pied flycatcher,** ***Ficedula hypoleuca***	15	1.862±0.570	0.740±0.238	15	5.763±1.421	2.307±0.654
**Song thrush,** ***Turdus philomelos***	4	1.222±0.586	1.260±0.678	4	7.157±2.045	7.389±2.607
**Blackbird,** ***Turdus merula***	6	0.939±0.307	1.353±0.525	6	4.881±1.233	7.013±2.147
**Fieldfare,** ***Turdus pilaris***	3	1.587±0.111	1.904±0.257	3	7.521±3.852	9.200±5.259
**Redwing,** ***Turdus iliacus***	4	1.168±0.376	1.009±0.276	4	5.509±1.543	4.745±0.996

Table S2. Dummy code for the two-level categorical variables included in the phylogenetic mixed models.

	**-0.5**	**0.5**
**Developmental mode**	altricial	precocial
**Migratory status**	resident	migratory
**Captivity**	wild	captive

Table S3. The proportion of variance in yolk THs explained by the extraction batch

Hormone	Parameter	Posterior mean [95% CI]
**T3**	concentration	0.10 [0.02, 0.25]
	total content	0.09 [0.01, 0.22]
**T4**	concentration	0.18 [0.03, 0.42]
	total content	0.14 [0.02, 0.32]

Table S4. Phylogenetic heritability calculated based on models including different fixed factors. The values for “Reported models” are the same as Table 2 in the main text.

	**T3 concentration**	**T3 content**	**T4 concentration**	**T4 content**
**Reported models**	0.84 [0.68, 0.95]	0.84 [0.71, 0.95]	0.60 [0.25, 0.87]	0.75 [0.54, 0.91]
**Removing “captivity”**	0.89 [0.79, 0.97]	0.85 [0.68, 0.96]	0.67 [0.31, 0.92]	0.81 [0.59, 0.95]
**No fixed factors**	0.90 [0.81, 0.96]	0.95 [0.89, 0.98]	0.75 [0.53, 0.92]	0.91 [0.81, 0.97]

Table S5. Estimated associations (posterior means and 95% credible intervals) between maternal yolk THs and key life-history traits.

Yolk hormone	Life-history trait	Model set	Posterior mean [95% CI]
T4 concentration	Developmental mode	1	0.562 [-0.426, 1.527]
	Migration	1	1.166 [0.436, 1.888]
	Body mass	1	-0.060 [-0.408, 0.288]
	Maximum lifespan	1	0.069 [-0.399, 0.542]
	Captivity	1	1.359 [0.418, 2.307]
	Incubation duration	2a	1.165 [-0.668, 3.001]
	Age at fledging	2a	-0.572 [-1.411, 0.265]
	Incubation duration	2b	1.329 [-0.207, 2.838]
	Age at fledging	2b	-0.565 [-1.381, 0.265]
	Clutch size	3	-0.391 [-1.188, 0.458]
	Number of clutches per year	3	-0.066 [-0.303, 0.173]
	Age at sexual maturity	4	0.089 [-0.176, 0.353]
	BMR	5	-0.055 [-1.087, 0.988]
	Growth rate	6	-1.041 [-2.743, 0.677]
T4 contents	Developmental mode	1	0.814 [0.278, 1.348]
	Migration	1	0.519 [0.147, 0.894]
	Body mass	1	0.478 [0.305, 0.653]
	Maximum lifespan	1	0.006 [-0.207, 0.217]
	Captivity	1	0.563 [0.070, 1.045]
	Incubation duration	2a	0.765 [-0.154, 1.673]
	Age at fledging	2a	-0.137 [-0.537, 0.261]
	Incubation duration	2b	1.151 [0.309, 1.987]
	Age at fledging	2b	-0.146 [-0.561, 0.270]
	Clutch size	3	-0.221 [-0.636, 0.192]
	Number of clutches per year	3	-0.075 [-0.194, 0.043]
	Age at sexual maturity	4	0.041 [-0.077, 0.163]
	BMR	5	-0.023 [-0.511, 0.464]
	Growth rate	6	-0.281 [-1.119, 0.545]

Table S5 (cont.). Estimated associations (posterior means and 95% credible intervals) between maternal yolk THs and key life-history traits.

Yolk hormone	Life-history trait	Model set	Posterior mean [95% CI]
T3 concentration	Developmental mode	1	0.680 [-0.337, 1.714]
	Migration	1	1.045 [0.358, 1.726]
	Body mass	1	-0.150 [-0.463, 0.162]
	Maximum lifespan	1	0.204 [-0.145, 0.558]
	Captivity	1	1.386 [0.487, 2.274]
	Incubation duration	2a	1.146 [-0.473, 2.776]
	Age at fledging	2a	-0.112 [-0.803, 0.588]
	Incubation duration	2b	1.380 [-0.039, 2.796]
	Age at fledging	2b	-0.118 [-0.803, 0.570]
	Clutch size	3	-0.025 [-0.726, 0.677]
	Number of clutches per year	3	0.025 [-0.179, 0.229]
	Age at sexual maturity	4	0.214 [0.028, 0.403]
	BMR	5	-0.051 [-0.909, 0.792]
	Growth rate	6	0.771 [-0.570, 2.098]
T3 contents	Developmental mode	1	0.841 [0.270, 1.412]
	Migration	1	0.572 [0.190, 0.954]
	Body mass	1	0.340 [0.166, 0.514]
	Maximum lifespan	1	0.095 [-0.100, 0.291]
	Captivity	1	0.708 [0.213, 1.208]
	Incubation duration	2a	0.858 [-0.051, 1.766]
	Age at fledging	2a	0.041 [-0.345, 0.431]
	Incubation duration	2b	1.227 [0.391, 2.068]
	Age at fledging	2b	0.038 [-0.367, 0.441]
	Clutch size	3	-0.094 [-0.050, 0.311]
	Number of clutches per year	3	-0.021 [-0.139, 0.097]
	Age at sexual maturity	4	0.132 [0.027, 0.237]
	BMR	5	0.009 [-0.504, 0.523]
	Growth rate	6	0.481 [-0.275, 1.225]

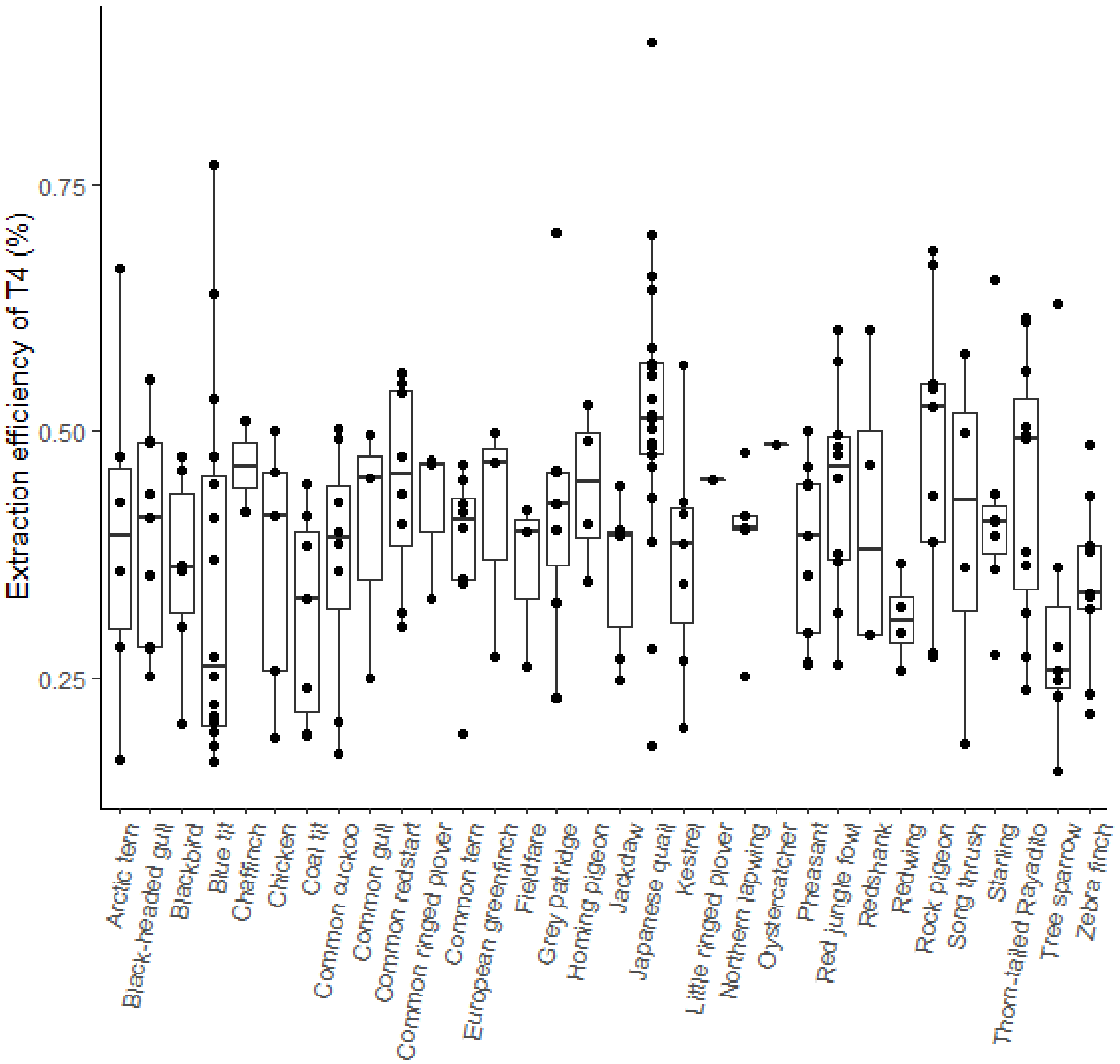

224 **Figure S1.** Recovery (i.e. extraction efficiency) of yolk T4 concentrations across species.

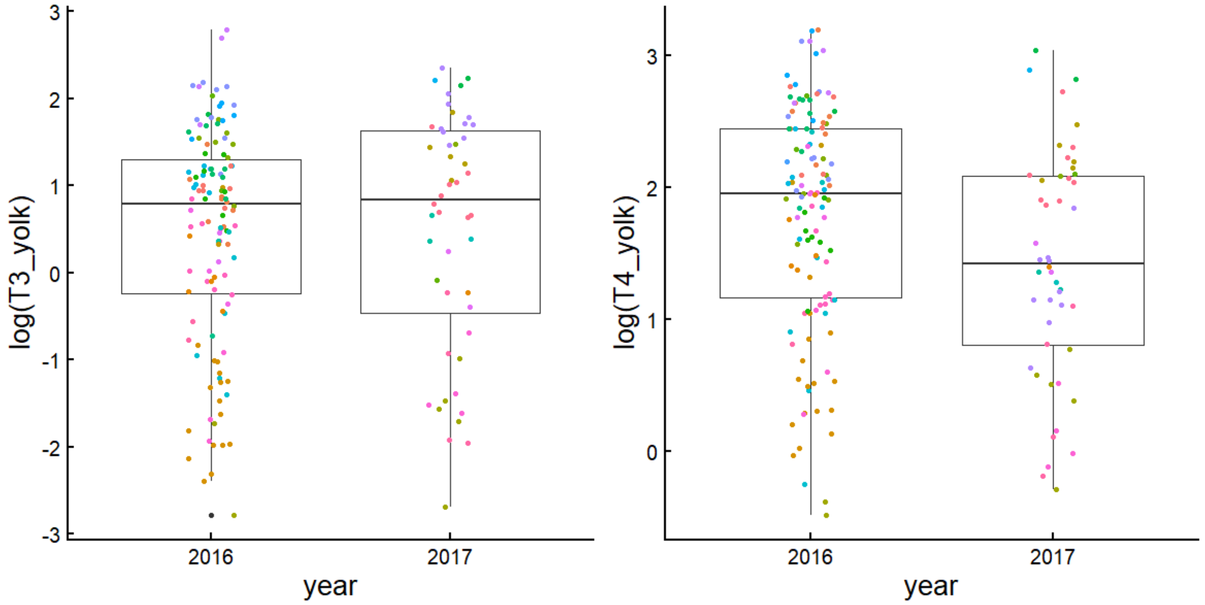

Figure S2. Yolk T3 and T4 concentrations (ln-transformed) in eggs collected in 2016 and 2017. Dots with different colours indicate different species.

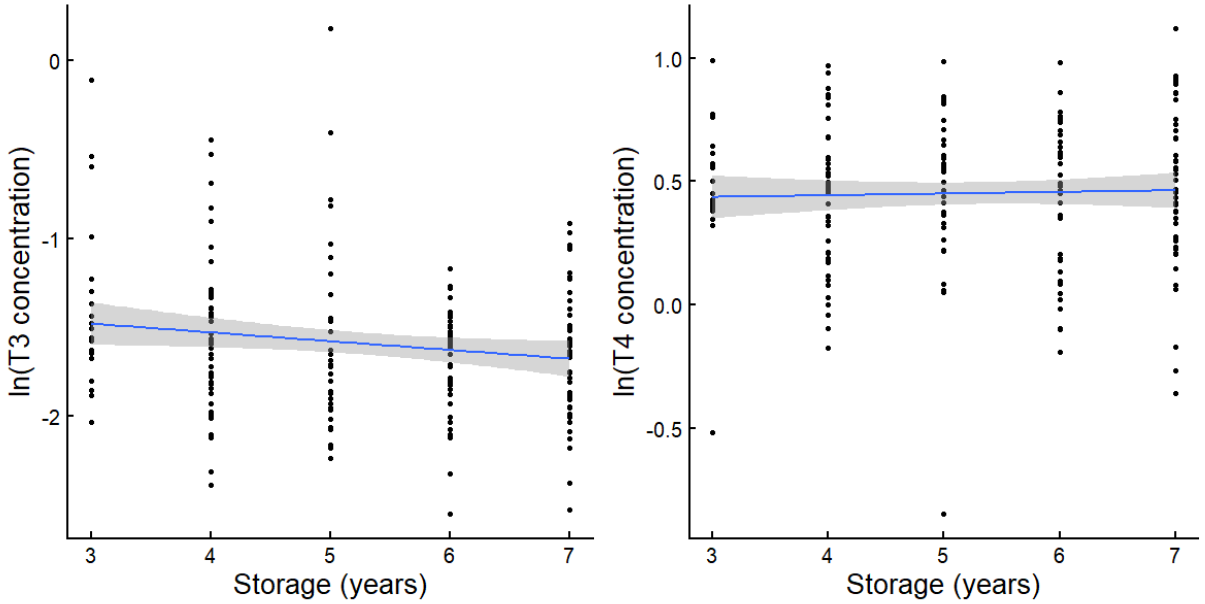

Figure S3. Linear change of yolk T3 (left) and T4 (right) concentration over 3-7 years of storage in great tit (*Parus major*) eggs. Shaded areas: 95% confidence intervals.

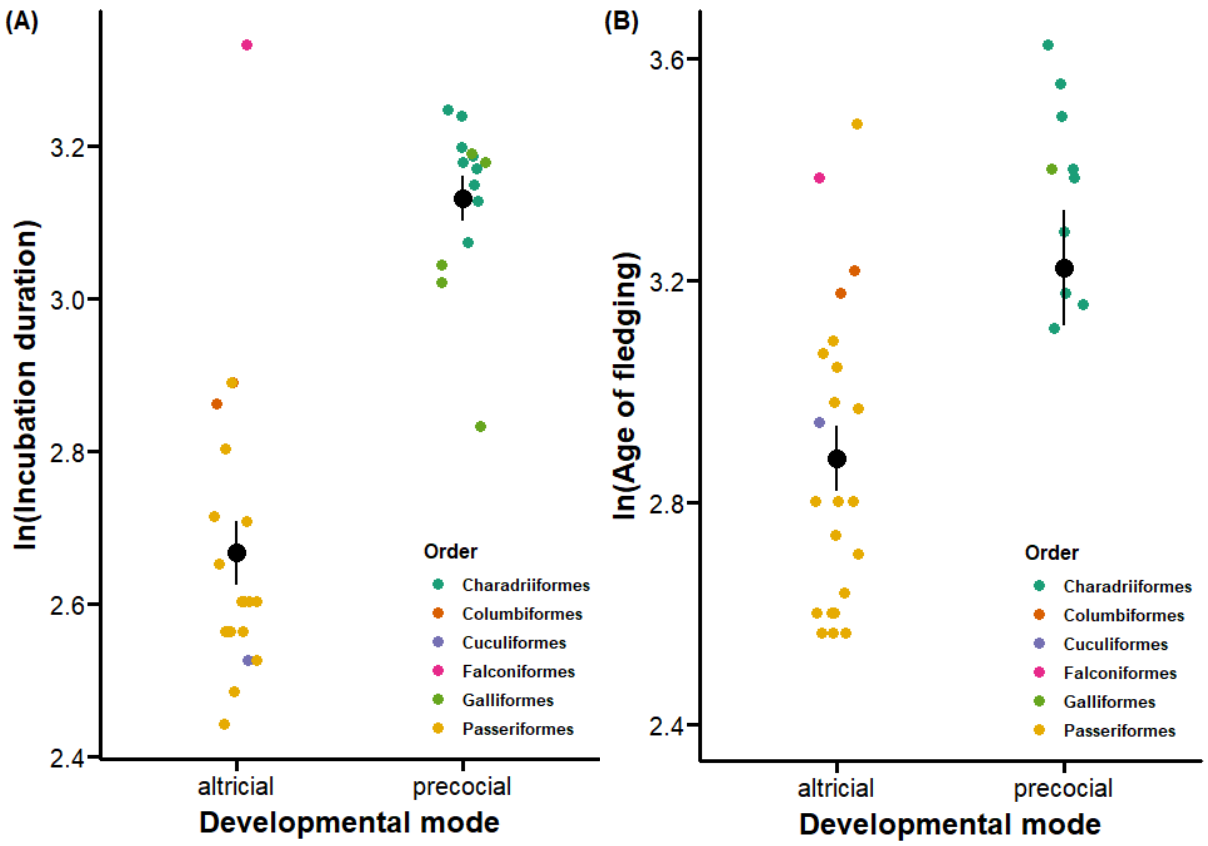

Figure S4. Differences in incubation duration and age of fledging between altricial and precocial species. Precocial species have clearly longer incubation duration (A, t=9.14, p<0.0001) and fledge at older ages (B, t=2.86, p=0.0010).

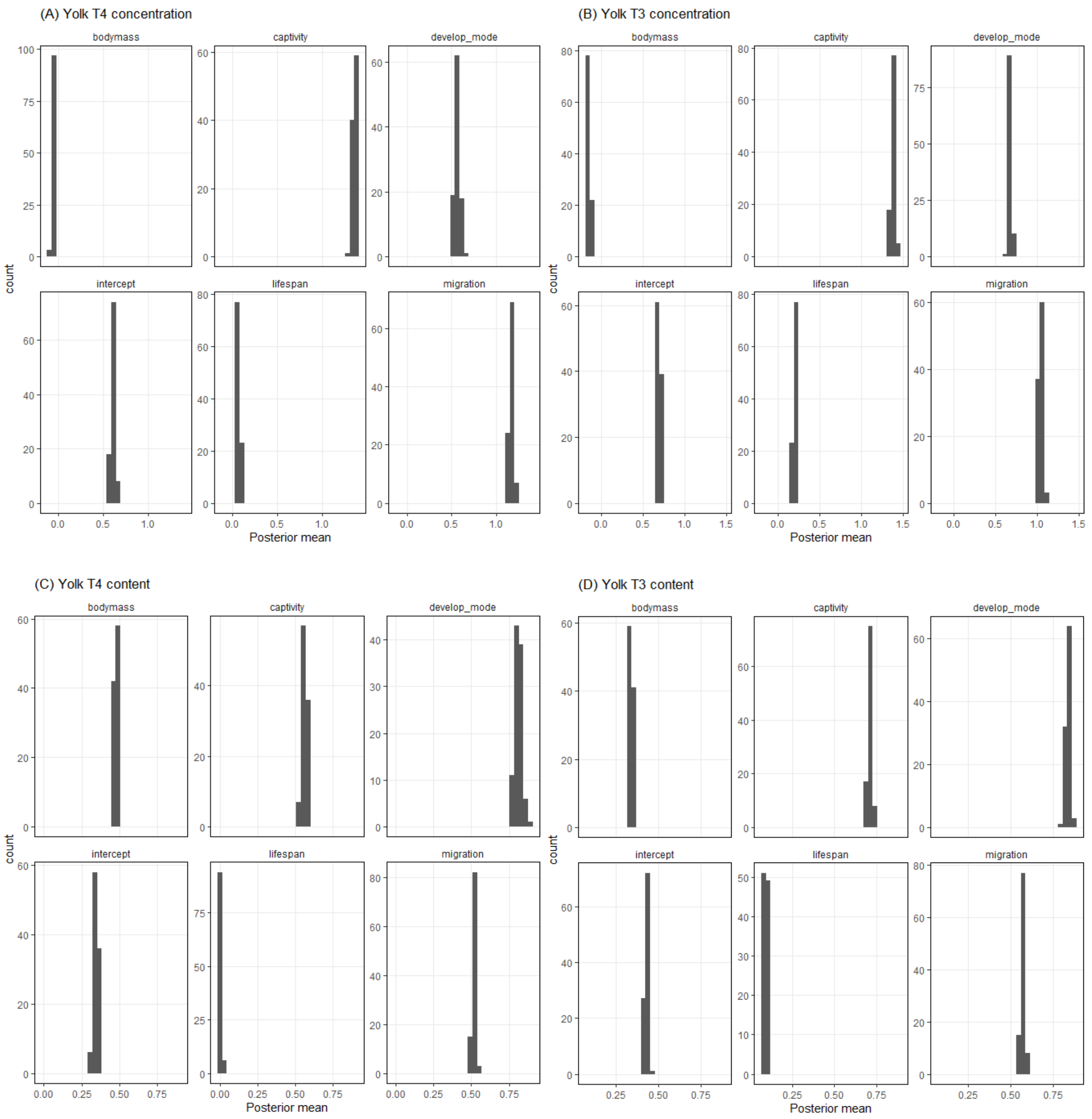

Figure S5. Histograms of posterior means for each life-history trait tested in model and data set 1 (see Table 1) across 100 possible phylogenetic trees from the Hackett backbone. All results were highly similar across different trees, indicated by the narrow range of the posterior means.

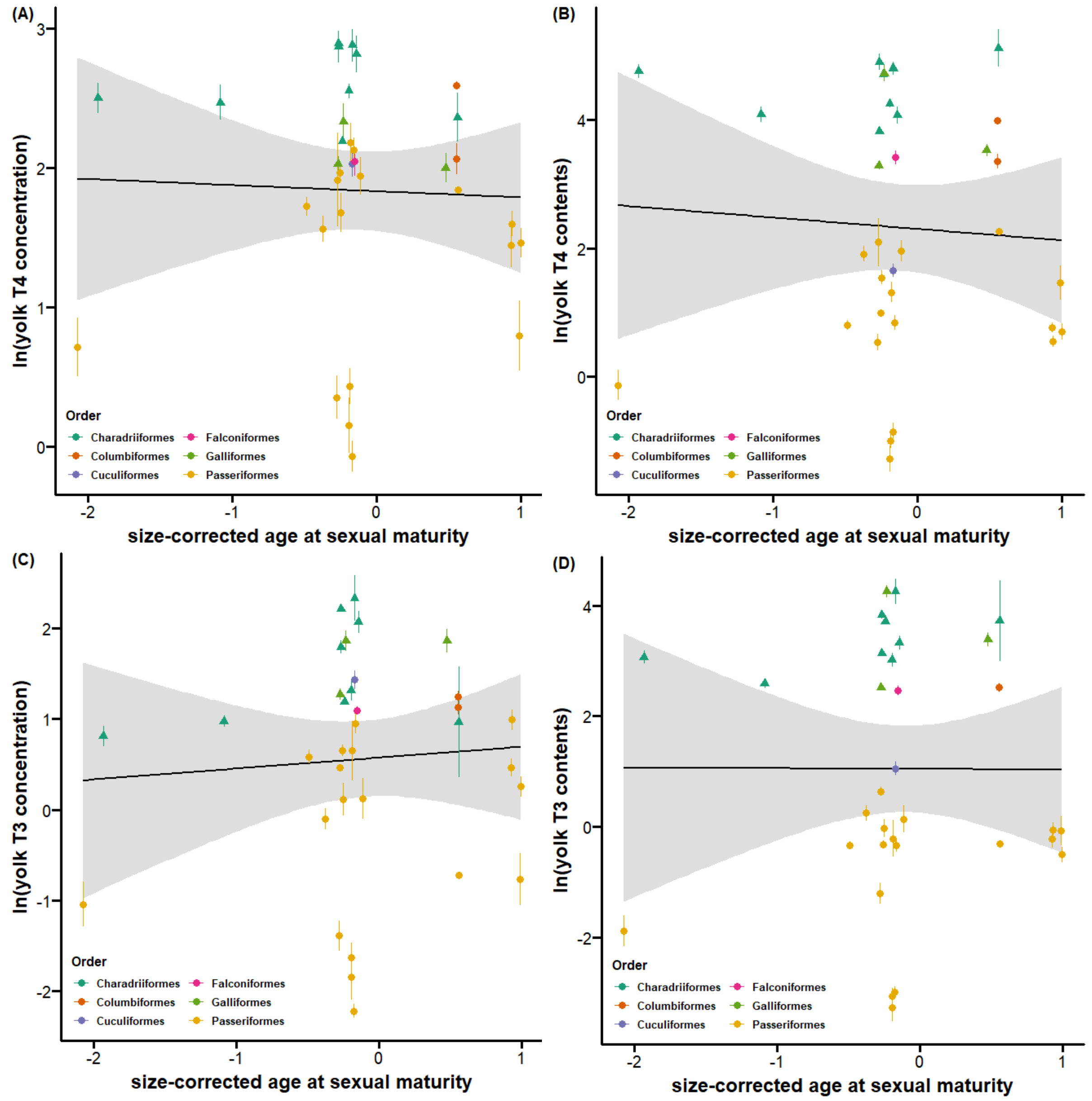

Fig. S6. Scatterplots of yolk THs and age at sexual maturity across species. Dots and triangles (±SE) represent altricial and precocial species, respectively. Black lines (shaded areas: 95% CI) represent the average correlation between incubation duration and yolk THs across all species.

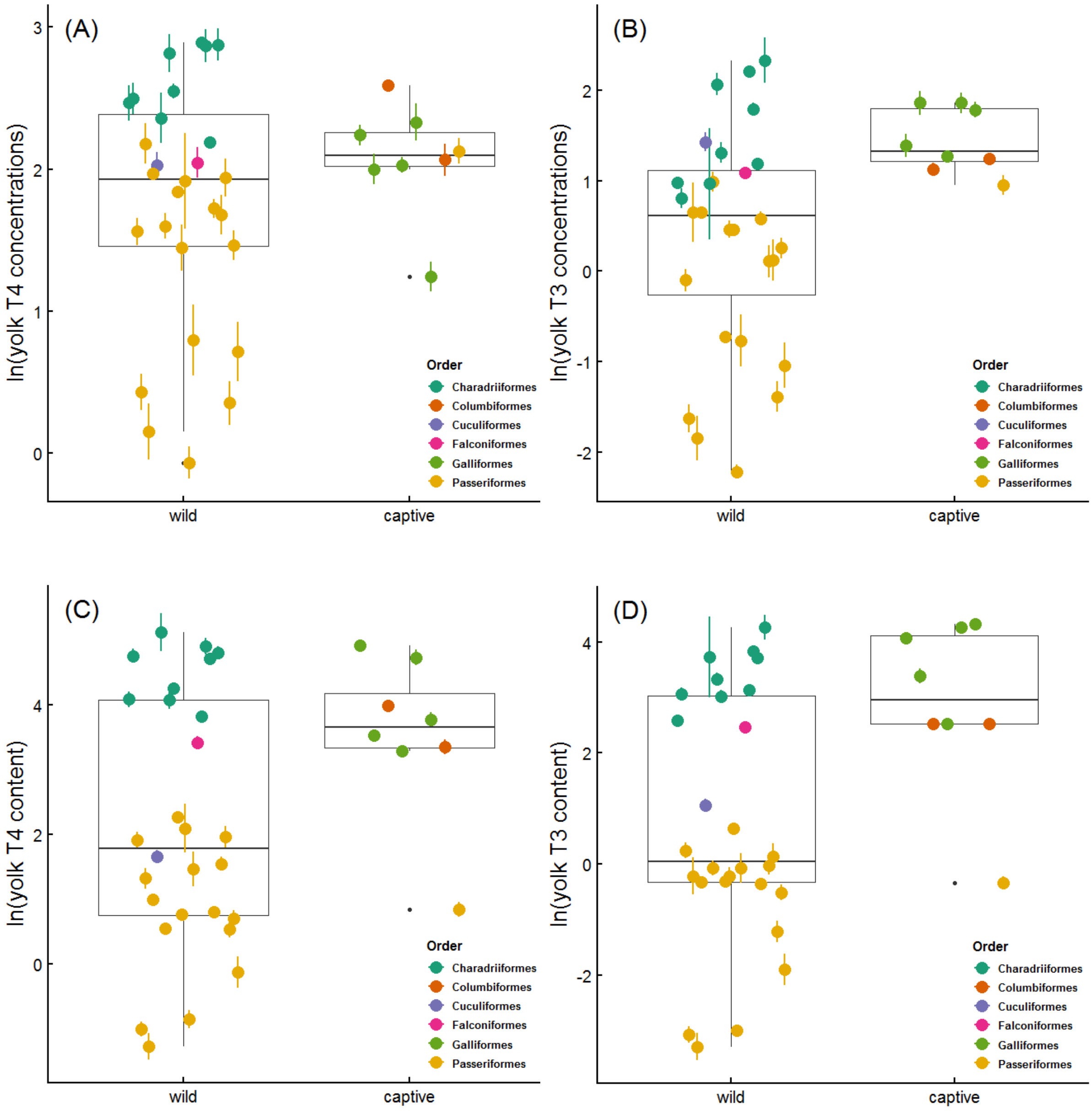

Figure S7. Boxplots of yolk TH concentrations (A, B, pg/mg, ln-transformed) and total contents (ng/yolk, ln-transformed) between captive and wild species. Boxplots represent the median (the middle line) and the first and the third quartiles (the box), and the whiskers extend to 1.5 times of the interquartile range. Colored dots represent species-specific means (±SE).

Cited References in supplementary methods and results

Barja, G. (2013). Updating the mitochondrial free radical theory of aging: an integrated view, key aspects, and confounding concepts. *Antioxi. Redox Signaling*, 19, 1420-1445.

Cramp, S. (1977-1994). Handbook of the Birds of Europe, the Middle East and North Africa. The Birds of the Western Palearctic. Vo. 1-9. Oxford University Press, Oxford, New York.

de Magalhães, J.P., Costa, J., Church, G.M. (2007). An analysis of the relationship between metabolism, developmental schedules, and longevity using phylogenetic independent contrasts. *J. Gerontol. Biol. Sci.*, 62A, 149-160.

Gil, D., Biard, C., Lacroix, A., Spottiswoode, C.N., Saino, N., Puerta, M., et al. (2007). Evolution of yolk androgens in birds: development, coloniality, and sexual dichromatism. *Am. Nat.* 169, 802-819.

Gorman, K.B., Williams, T.D. (2005). Correlated evolution of maternally derived yolk testosterone and early developmental traits in passerine birds. *Biol. Lett.* 1, 461-464.

Hackett, S.J., Kimball, R.T., Reddy, S., Bowie, R.C.K., Braun, E.L., Braun, M.J. *et al.* (2008). A phylogenomic study of birds reveals their evolutionary history. *Science,* 320, 1763-1768.

Hackl, R., Bromundt, V., Daisley, J., Kotrschal, K., Möstl, E. (2003). Distribution and origin of steroid hormones in the yolk of Japanese quail eggs (*Coturnix coturnix japonica*). *J. Comp. Physiol. B*, 173, 327-331.

Hadfield, J.D., Nakagawa, S. (2010). General quantitative genetic methods for comparative biology: phylogenies, taxonomies and multi-trait models for continuous and categorical characters. *J. Evol. Biol.,* 23, 494-508.

Healy, K, Guillerme, T., Finlay, S., Kane, A., Kelly, S.B.A., McClean, D. *et al.* (2014). Ecology and mode-of-life explain lifespan variation in birds and mammals. *Proc. R. Soc. B*, 281, 20140298.

Hsu, B.-Y., Verhagen, I., Gienapp, P., Darras, V.M., Visser, M.E., Ruuskanen, S. (2019). Between- and within-individual variation of maternal thyroid hormone deposition in wild great tits (*Parus major*). *Am. Nat.*, 194, E96-E108.

Ives, A.R., Midford, P.E., Garland, T., Jr. (2007). Within-species variation and measurement error in phylogenetic comparative methods. *Syst. Biol.*, 56, 252-270.

Lipar, J.L., Ketterson, E.D., Nolan V., Jr., Casto, J.M. (1999). Egg yolk layers vary in the concentration of steroid hormones in two avian species. *Gen. Comp. Endocrinol.*, 115, 220-227.

MacLean, B., Tomazela, D.M., Shulman, N., Chambers, M., Finney, G.L., Frewen, B. *et al.* (2010). Skyline: an open source document editor for creating and analyzing targeted proteomics experiments. *Bioinformatics,* 26, 966-968.

McKechnie, A.E., Freckleton, R.P., Jetz, W. (2006). Phenotypic plasticity in the scaling of avian basal metabolic rate. *Proc. R. Soc. B,* 273, 931-937.

McNab, B.K. (2009). Ecological factors affect the level and scaling of avian BMR. *Comp. Biochem. Physiol. A,* 152, 22-45.

Moreno, J., Merino, S., Vásquez, R.A., Armesto, J.J. (2005). Breeding biology of the thorn-tailed rayadito (Furnariidae) in south-temperate rainforests of Chile. *Condor*, 107, 69-77.

Myhrvold, N.P., Baldridge, E., Chan, B., Sivam, D., Freeman, D.L., Ernest, S.K.M. (2015). An amniote life-history database to perform comparative analyses with birds, mammals, and reptiles. *Ecology,* 96, 3109.

Nagy, K.A., Girard, I.A., Brown, T.K. (1999). Energetics of free-ranging mammals, reptiles, and birds. *Annu. Rev. Nutr.*, 19, 247-277.

Nakagawa, S., Schielzeth, H. (2010). Repeatability for Gaussian and non-Gaussian data: a practical guide for biologists. *Biol. Rev.*, 85, 935-956.

Quirici, V., Hammers, M., Botero-Delgadillo, E., Cuevas, E., Espíndola-Hernández, P., Vásquez, R.A. (2019). Age and terminal reproductive attempt influence laying date in the thorn-tailed rayadito. *J. Avian Biol.* 50, e02059

Revell, L.J. (2012). phytools: an R package for phylogenetic comparative biology (and other things). *Methods Ecol. Evol.,* 3, 217-223.

Ruuskanen, S., Darras, V.M., de Vries, B., Visser, M.E., Groothuis, T.G.G. (2016a). Experimental manipulation of food availability leads to short-term intra-clutch adjustment in egg mass but not in yolk androgen or thyroid hormones. *J. Avian Biol.,* 47: 36-46.

Ruuskanen, S., Groothuis, T.G.G., Schaper, S.V., Darras, V.M., de Vries, B., Visser, M.E. (2016b). Temperature-induced variation in yolk androgen and thyroid hormone levels in avian eggs. *Gen. Comp. Endocrinol.*, 235, 29-37.

Ruuskanen, S., Hsu, B.-Y., Heinonen, A., Vainio, M., Darras, V.M., Sarraude, T., et al. (2018). A new method for measuring thyroid hormones using nano-LC-MS/MS. *J. Chromatogr. B,* 1093-1094, 24-30.

Schwabl, H., Palacios, M., Martin, T.E. (2007). Selection for rapid embryo development correlates with embryo exposure to maternal androgens among passerine birds. *Am. Nat.* 170, 196-206.

Starck, J.M., Ricklefs, R.E. (1998). *Avian Growth and Development: Evolution within the Altricial-Precocial Spectrum*. Oxford University Press, New York, NY.

Tacutu, R., Craig, T., Budovsky, A., Wuttke, D., Lehmann, G., Taranukha, D., et al. (2013). Human ageing genomic resources: Integrated databases and tools for the biology and genetics of ageing. *Nucleic Acids Res.,* 41, D1027-D1033.

Tricola, G.M., Simmons, M.J.P., Atema, E., Boughton, R.K., Brown, J.L., Dearborn, D.C., et al. (2018). The rate of telomere loss is related to maximum lifespan in birds. *Phil. Trans. R. Soc. B*, 373, 20160445.

Vágási, C.I., Vincze, O., Pătraş, L., Osváth, G., Pénzes, J., Haussmann, M.F., et al. (2019). Longevity and life history coevolve with oxidative stress in birds. *Funct. Ecol.,* 33, 152-161.

van Herck, S.L.J., Geysens, S., Delbaere, J., Tylzanowski, P., Darras, V.M. (2012). Expression profile and thyroid hormone responsiveness of transporters and deiodinases in early embryonic chicken brain development. *Mol. Cell. Endocrniol.*, 349, 289-297.
